# Beyond Small Molecules: Orchestrating Cell Fate with Engineered Water-Soluble Membrane Proteins

**DOI:** 10.3390/biom16040546

**Published:** 2026-04-08

**Authors:** Sebastian Valencia-Amores, Israel Davila Aleman, Timothy G. Jenkins, Dario Mizrachi

**Affiliations:** 1 Department of Cell Biology and Physiology, Brigham Young University, Provo, UT 84602, USA; 2 Department of Biomedical Sciences, Noorda College of Osteopathic Medicine, Provo, UT 84606, USA

**Keywords:** water-soluble membrane proteins, regeneration, cell differentiation

## Abstract

The potential of water-soluble membrane proteins (wsMPs) has not been fully realized. In this article, we exploit the nearly identical functionality of wsMPs with their membrane-bound counterparts and show that we can create water-soluble membrane proteins that incorporate into the plasma membranes of cells and alter their fate. As a proof of concept, we demonstrate the functional properties of water-soluble engineered pore-forming proteins, K^+^ ionic channels (MthK), and constitutively active GPCRs—among them frizzled receptors—both in vitro and in vivo. We call this method in vivo deployment of recombinant viable MPs, iDRIVE. Furthermore, we demonstrate that our strategy mediates the unidirectional insertion of MPs into the plasma membrane, and through constitutively active receptors, we present evidence for similar signaling pathway activation between small molecules and our water-soluble proteins using model phenotypes and molecular signaling assays. We present three examples where wsMPs are functional in dictating cellular fate, both in vitro and in vivo. Lastly, we show the induction of similar differential methylation via the activation of the Wnt signaling pathway using the conventional small molecule agonist, CHIR99021, or our wsFrizzled receptors (iDRIVE-FZD) in human embryonic kidney (HEK 293) embryoid spheroids (ESs). Additionally, we show that Wnt activation via wsFrizzled receptors results in even more biologically relevant epigenetic changes than via the small molecule CHIR99021. Future work will employ iDRIVE to differentiate stem cells in the production of research and clinically relevant organoids.

## 1. Introduction

Membrane proteins (MPs), including the subset of integral membrane proteins, constitute 20–30% of all open reading frames (ORFs) in the genome of living organisms [1]. Despite their relative abundance and high relevance for cellular function, they remain a complex subject in research [2]. In 1935, Danielli and Harvey [3] published the first model that suggested, based on surface tension studies, that cell membranes consist of a lipid bilayer with protein layers on both the inner and outer surfaces. Indeed, they were the first to acknowledge the presence of proteins and their role in cellular membranes. In 1978, Glycophorin was the first MP to be characterized by resolving its primary sequence [4]. Nowadays, the study of MPs, which are naturally of low expression, is often carried out using recombinant systems [5], and solutions to the typical challenges associated with working with MPs are the result of trial and error, e.g., selecting the correct host, protein purification, detergent selection for purification and maintenance of function, yields, etc. [6]. Moreover, MPs serve as critical drug targets across various medical fields but remain large obstacles in drug discovery due to the complexities involved in producing MPs in functional forms [7]. A significant portion of predicted disease-causal proteins have proven resistant to targeting by traditional modalities. In that sense, MPs have largely been classified as undruggable targets [8].

With the advent of synthetic biology and genetic tools to synthesize DNA and RNA, investigators have renewed their efforts to solve the MP challenge and, since the early 2000s, have tried to create water-soluble MPs. However, it was much later that a definition for water-soluble membrane proteins (wsMPs) was given, in which wsMPs were categorized as membrane-associated proteins that remain stably soluble in aqueous buffer (without detergents or lipids) while still retaining their ability to associate with, or originate from, biological membranes [9]. In the year 2002, the laboratory of Steven Sligar published a method named Nanodiscs [10] to describe a strategy that relies on a phospholipid bilayer encircled by two copies of an amphipathic helical protein termed the membrane scaffold protein (MSP). A variety of MSPs, with varying lengths and tags, were engineered from the human Apo-AI sequence to generate monodisperse and homogeneous discoidal bilayer structures [11]. In this approach, the membrane protein target is transiently solubilized with a detergent in the presence of phospholipids and an encircling amphipathic helical protein belt, the MSP. Thus, MP production is independent of the nanoparticle and faces similar challenges as recombinantly produced and purified MPs [12]. The detergent-purified MPs are then incubated and partitioned in the nanoparticle for further functional and structural studies [13].

In 2009, Knowles and colleagues [14] presented a report that indicated that bilayer disks formed by phospholipids and the styrene maleic anhydride copolymer preserve the functional and structural integrity of α-helical and β-barrel transmembrane proteins. The Styrene Maleic Acid Lipid Particle (SMALP) technology allows for the detergent-free extraction of membrane proteins directly from plasma membrane and solubilizes membrane bilayers into ~10–90 nm discs. This method avoids potentially destabilizing detergents; preserves native protein structure; and keeps membrane proteins in a lipid-rich, native-like environment [15]. Nevertheless, like nanodiscs, their success depends on the ability to produce MPs, but unlike nanodiscs, it does not require detergents or partition of the MP from the detergent micelle to the SMALP.

More recently, protein engineering tools have been applied to solve the production challenges of MPs [16]. Making water-soluble membrane proteins is a new approach achieved by two separate groups in the last 10 years [17,18]. In 2018, Zhang and colleagues [18] published the QTY model, in which the hydrophobic regions of a membrane protein are mutated to favor their interaction with water. In their seminal publication, G-protein coupled receptors (GPCRs) were used as proof of concept for the QTY model. The detergent-free QTY variants maintain a stable structure and retain ligand-binding activities [18]. In contrast, Mizrachi and colleagues achieved integral membrane protein water solubilization by using fusion proteins; this model was tested on membrane proteins of different topology and structures [17]. This method, called SIMPLEx (solubilization of integral membrane proteins with high levels of expression), generated triple fusion consisting of a soluble protein fused to the integral membrane protein and ultimately fused to Apolipoprotein AI (Apo AI), an amphipathic protein that supports the hydrophobic domain of the membrane protein and prevents destabilizing interactions with water. SIMPLEx allows the triple fusion to be expressed in the cytosol of hosting cells. In the initial 2015 report, SIMPLEx was reported to produce membrane proteins with active conformations, as demonstrated through the triple fusion with EmrE (ethidium multidrug resistance protein E), an *E. coli* transport protein, where affinity for its natural ligands was established but not its transport function [17]. More recently, SIMPLEx was demonstrated to produce fully functional membrane proteins [19].

Finally, a 2025 preprint by the laboratory of Dr. David Baker, Institute for Protein Design (https://www.ipd.uw.edu/, accessed on 15 March 2026), described a similar strategy as SIMPLEx to design wsMPs using de novo protein WRAPs (Water-soluble RF-diffused Amphipathic Proteins) that surround the lipid-interacting hydrophobic surfaces, rendering them stable and water-soluble without the need for detergents [20]. The data presented indicate WRAPs can be designed from α-helices or β-sheets [20]. WRAPs should be broadly useful for facilitating biochemical and structural characterization of MPs.

The initial goal of generating wsMPs was to improve the structural characterization of membrane proteins and aid in the structure–function studies of these elusive targets. We propose that the potential of wsMPs has still not been fully realized. Given that wsMPs produced by SIMPLEx were shown to retain a native-like functionality, understanding structure–function relationships is not the only use for them. Here, we propose that wsMPs may be used in functional studies in vitro and in vivo. In our present work, we modify the order of the triple fusion protein suggested by SIMPLEx and describe a method we named iDRIVE (in vivo deployment of recombinant viable MPs) and show that our technique can produce soluble and functional integral membrane proteins capable of incorporating into plasma membranes of in vitro and in vivo models and elicit targeted cellular changes. More specifically, we show that iDRIVE can produce soluble and functional pore-forming-proteins (PFPs), ion channels, and GPCRs that can influence and dictate cellular and organismal phenotypic fates. Furthermore, our technique provides a new paradigm that allows the functional study of mutations or truncations of MPs without the use of gene editing techniques. Our methodology presented herein, iDRIVE, provides a new paradigm that addresses the undruggable MP target problem by enabling the target production of soluble, functional, and biologically active signaling MPs.

## 2. Materials and Methods

### 2.1. Materials

Bacterial cells were obtained from New England Biolabs (Ipswich, MA, USA). Mammalian cells HEK 293 and C2C12 were obtained from ATCC (Manassas, VA, USA). Planaria were purchased from Carolina Biologic Supply (Burlington, NC, USA). Plasmids and DNA synthesis were obtained through TWIST Biosciences (San Francisco, CA, USA). All accession numbers of the proteins used in this work are in Table S1. Following that table, the constructs are defined by their amino acid sequence. All amino acid sequences were codon-optimized for K12 *E. coli* prior to engaging TWIST for plasmid synthesis. Chemicals (CHIR99021, Retinoic Acid) were obtained through Sigma Aldrich (St. Louis, MO, USA). Media for mammalian cells and bacterial cells culture, and general salts and buffers were obtained from Genesee Scientific (El Cajon, CA, USA).

### 2.2. Planarian Husbandry

Planaria were kept at room temperature (in our laboratory, this corresponds to 21 °C) under conditions of darkness and fresh water (Instant Ocean salt, 0.5 g/L; Spectrum Brands Inc., Middleton, WI, USA). Planaria were fed beef liver once a week. Prior to the experiments, the planaria were starved for 7 days. Incisions and cuts were performed with scalpels on a filter paper-covered cold pack. Fragments were separated and kept in 6-well plates with 2 mL of Instant Ocean water. Treatments consisted of 0.5 µM of iDRIVE or a control (OspA-ApoAI-GFP-CPP), which was replaced with new ocean water every 8 h. And, for routine maintenance, the water was replaced at least once or twice a week depending on the planaria population numbers and after feeding. For more details regarding planaria husbandry, see the Dean and Duncan 2020 review [21]. Recombinant planaria Frizzled receptor Frizzled4mSx2 (https://www.uniprot.org/uniprotkb/A0A159X4Q5/entry, accessed on 22 March 2026) or Frizzled1/2/7mSx2 (https://www.uniprot.org/uniprotkb/T1E1C6/entry, accessed on 22 March 2026) were used in the regeneration experiments.

### 2.3. Core Facilities

Brigham Young University (Provo, UT, USA) provided the electron microscopy data (https://microscopy.byu.edu/home, accessed on 15 January 2026). Core facilities at the University of Utah (https://cores.utah.edu/, accessed on 3 March 2026) (Salt Lake City, UT, USA) provided the epigenetics analysis.

### 2.4. HEK 293 Cell Culture and Spheroids

Tissue culture protocols followed the classical methods using DMEM and 10% FBS. Regarding spheroid formation, we followed Iuchi and colleagues’ protocols from their publication in 2020 [22].

### 2.5. Protein Expression and Purification

Our protocols for protein expression and purification have been published previously [17]. Briefly, we employed Terrific broth as a medium for cells to grow at 37 °C. IPTG induction commenced when OD_600_ was 0.8–1. The temperature was then lowered to 16 °C for a period of 16–18 h. All our protein constructs contained a 6xHis tag and were purified using NiNTA [17]. For initial protein expression anti-GFP antibody (B-2), 1:1000 (Santa Cruz Biotechnologies, Santa Cruz, CA, USA) and anti-6xHis monoclonal antibody (MA1-135; Invitrogen, Carlsbad, CA, USA) were employed at a dilution of 1:20,000. Final steps toward developing the image were carried out using the LI-COR system (Lincoln, NE, USA). TATAx3: We prepared the TATAx3 protein to be expressed without any other modifications besides a 6xHis tag in a pET28a plasmid.

### 2.6. Confocal Microscopy

*E. coli* expressing proteins with N-terminally fused GFP were harvested and diluted, 1:100, in Luria–Bertani medium. Poly-lysine (Sigma-Aldrich)-coated slide glass was used to mount the cells. A cover glass was placed over the cells and sealed in place with clear nail polish. Cells were imaged within 1 h of their preparation with a Zeiss LSM710 confocal microscope (Zeiss, Oberkochen, Germany) equipped with a ×100 oil immersion objective.

### 2.7. Negative Staining

Freshly purified OspA-TATAx3 was prepared at different concentrations (0.5, 0.25, 0.1, and 0.05 mg mL^−1^) for negative staining by applying a 5 μL protein drop to a carbon-coated grid (300-mesh copper grid) for 2 min and blotting with filter paper to remove excess solution. A second solution of 1.5% uranyl acetate was immediately applied for another 2 min. Dried grids were examined using an FEI Tecnai 12 Spirit Twin electron microscope. Twenty fields for each sample concentration were randomly selected and photographed at different magnification levels. Image analysis was then performed with ImageJ software (Version 1.54r 25 September 2025).

### 2.8. Biological SAXS

Data were collected at the Cornell High Energy Synchrotron Source (CHESS) G1 station in Ithaca, New York. Protein samples of ΔspMBP-EmrE-Apo AI* were exposed to a 250 × 250 μm beam of 9.968 keV X-ray. Sample preparation included centrifugation at 30,000× *g* for 30 min and filtration to remove any aggregates. Samples (30 μL) were loaded and oscillated in the beam using an automated system with a plastic chip-based sample cell (2 mm path) and polystyrene X-ray transparent windows. The sample cell and X-ray flight path were placed under vacuum to reduce background scattering. Scattering patterns were captured on a Pilatus 100K-S detector (Dectris, Baden, Switzerland) at a 1504 mm distance. The exposure time was 5 s for each image, and 10 images were recorded for each sample. All mathematical manipulations of the data (azimuthal integration, normalization, averaging, and buffer subtraction) as well as error propagation were carried out using the RAW software [23]. The range of momentum transfer was calculated to be 0.0068 < q = 4π sin(θ)/λ < 0.28 Å^−1^, where 2θ is the scattering angle and λ = 1.257 Å is the X-ray wavelength. Dimer and tetramer samples were run at a range of concentrations (0.3, 0.6, 1.0, 2.0, 5.0, and 10 mg  mL^−1^) to evaluate for possible concentration effects. Molecular weight estimated from a lysozyme standard (3.5 mg  mL^−1^, 50 mM NaOAc, 50 mM NaCl pH 4.0) agreed with our expectations within error. Radius of gyration (Rg) was calculated using both the Guinier approximation and the inverse Fourier transform methods as implemented in the GNOM-ATSAS 2.3 package by D. Svergun EMBL-Hamburg. The pair distance distribution function P(r) was calculated using the GNOM program [24]. The maximum dimension of the particle, Dmax, was estimated based on the goodness of the data fit and smoothness of the decaying tail. The GNOM output file for the dimer was used as input to DAMMIF [25] to perform ab initio shape reconstruction without imposing any symmetry. The 20 reconstructed bead models were superimposed and averaged using DAMAVER in automatic mode. The mean NSD was 0.636 ± 0.047 (*n* = 20), where an NSD value <1 indicates close agreement between different reconstructed models.

### 2.9. Planar Bilayer

Planar lipid bilayer electrophysiology recordings were performed as described by Zakharian, using painted bilayers across a Teflon aperture and symmetrical salt solutions unless otherwise indicated [26]. Briefly, 100 µL of PEPC in a ratio of 7:3 (10 mg/mL) and 15 µL of 0–40 mol% cholesterol (Avanti Polar Lipids) were prepared using glass pipettes. Subsequently, the solvent was allowed to evaporate for 10 min under nitrogen gas, and the membrane was diluted in 50 µL of decane. Lipid bilayers were painted on custom cup apertures of different diameters, 150–400 µm. Membrane integrity was assessed using capacitance measurements. The membrane was repainted if Cm < 50% of the maximum expected value based on hole diameter or if Gm was irregular or greater than 10 pA leak current. Voltages were alternated between 100 mV and −100 mV across the cis–trans chambers with an intermittent duration of 1–2 min. For quality control, conductance was monitored for 10–20 min before any protein was added (Figure S5). Alternating potentials were applied until the first conductance event was observed. Furthermore, the cis chamber was continuously mixed using a small magnetic stir bar. Data were collected with a current low-pass filter at 1–2 kHz and sampled at 10 kHz. Proteins: OspA-ApoAI-TATAx3-CPP (0.05–0.5 µM) or OspA-ApoAI-MthK-CPP (0.07–0.4 µM) was added to the cis chamber, and conductance was measured. Protein concentrations were sequentially increased if no conductance event occurred. TATAx3 buffer solution: Both the cis and trans chambers were filled with 1 mL of 100 mM KCl and 20 mM Tris-HCl at pH 7.4, and 0.2 mM MgCl_2_ was supplemented to stabilize the bilayer and aid in protein fusion. MthK buffer solution: Both the cis and trans chambers were filled with 1 mL of 150 mM KCl, 20 mM MgCl_2_, 15 mM Tris at pH 8.02, and 450 mM of sucrose, as previously described [27]. Protein concentrations: Protein molarity concentrations were estimated using known purified protein concentrations (spectrophotometry) and protein weight using the online calculator (https://protein-calculator.org/protein-weight-calculator-kda.php, accessed on 28 February 2026) and converted to molarity using the bioline calculator (https://www.bioline.com/media/calculator/01_04.html, accessed on 28 February 2026). Free Ca^2+^: Free solution calcium was estimated using the ucdavies free ion online calculator with the following parameters: pH, 8.02; temperature, 22 °C; ionic contribution, 0.213 based on the MthK buffer solution with 1 mM CaCl_2_ and 2 mM EDTA. (https://somapp.ucdmc.ucdavis.edu/pharmacology/bers/maxchelator/webmaxc/webmaxcS.htm, accessed on 28 February 2026).

### 2.10. Differential Methylation Analysis

Human embryonic kidney (HEK 293) cells were treated every 12 h for 24 h with either 8 μM CHIR99021 + 10 µM Retinoic acid (RA) or 0.5 μM constitutively active human FZD7 (cahFZD7)+ 10 µM RA or 10 µM RA control with 2 samples per group. Samples were frozen until they were run on the Illumina Human MethylationEPIC BeadChip Version 2 array. Bioinformatic analyses were performed in R v4.5.1 using an aarch64-apple-darwin20 platform. Packages were installed using BiocManager v3.22 (https://bioconductor.github.io/BiocManager/, accessed on 19 January 2026) or directly from CRAN. A seed of 5126 was used to ensure reproducible results. For analysis metadata and package versions, see Figure S1. minfi [28] was used to import raw idat files into an RGChannelSetExtended object. Quality control: The *p*-values of failed probe positions were calculated with the minfi::detectionP function. Probes with *p*-val > 0.01 were considered to have “failed.” Sample quality control was performed by filtering samples that had over 5% “failed” probes. Similarly, probe filtering was performed by removing probes that “failed” in over 10% of the samples. Comprehensive data quality control was assessed with the minfi::qcReport function (Figures S8 and S9). Data processing: RGChannelSet objects were normalized using the minfi::preprocessNoob function with the “single” dyeMethod and dyeCorrection = TRUE parameters, and default parameters, unless otherwise specified. Beta and M-values were obtained using the minfi::getBeta and the minfi::getM functions, respectively. Annotation: Probes were annotated using the minfi::getAnnotation function with default parameters. CpG M-values were annotated using the DMRcate package [29] through the cpg.annotate function with the following parameters—datatype = “array”, arraytype = “EPICv2”, what = “M”, analysis.type = “differential”, contrasts = TRUE, and fdr = 0.05—along with design and contrast matrices used for differential methylation, described later. Cross reactive probes: Annotated probes that are known to have SNPs in their body, in their CpG sites, or at the single base extension position or are sex-specific were excluded from the analysis. Only probes with complete data (stats::complete.cases) across all samples were retained. Outlier detection: PCA analyses were performed, resulting in 5 PCs explaining all variance. The Mahalanobis distance was calculated on the 5 PCs, with each PC mean as the Mahalanobis center. The outlier threshold was defined using the qchisq value with probability 0.975 and 5 degrees of freedom. Differential methylation: A design matrix was built on the different treatment categories (RA control, CHIR + RA, and iDRIVE + RA). Differentially methylated CpGs were calculated using the limma [30] lmFit function followed by Empirical Bayes moderation to improve variance estimate reliability, using the limma::eBayes function on the M-values and the contrasts: CHIR+RA-RA control, iDRIVE + RA-RA control, and CHIR + RA-iDRIVE + RA. Differential methylation Regions: Differentially methylated regions were assessed with the DMRcate::dmrcate function using lambda = 1000, C = 2, a pcutoff = 0.01, and default parameters unless otherwise specified. Genomic ranges were extracted using the DMRcate:: extractRanges function using the “hg38” genome. Region plots: Differentially methylated regions’ Beta values were plotted using the DMRcate::DMR.plot function with the arguments genome = “hg38”, flank = 5000, heatmap = TRUE, and extra.ranges = NULL. Regional methylation values: M-values or Beta values were averaged across replicates by treatment group for each probe. Then, the average M or Beta DMR value was calculated by taking the mean (M or Beta) value of their corresponding probes. Graphics: ggplot2 (https://ggplot2.tidyverse.org/, accessed on 2 April 2026) or ggforce was used for bar plots and sina plots, ggpubr (https://rpkgs.datanovia.com/ggpubr/, accessed on 2 April 2026) to plot *p*-value statistics, eulerr::euler for the Venn diagram, and heatmaps using the package ComplexHeatmap [31] to plot Heatmaps. Hierarchical clustering of heatmap probes and samples was performed with their respective “Manhattan” distances using the “average” linkage method. Functional enrichment: DMRs across comparison were uploaded to the web-based GREAT tool by Stanford (http://great.stanford.edu/public/html/, accessed on 22 March 2026) in BED format using the GRCh38 genome and tested against the whole genome for background regions.

### 2.11. Statistics

Unless otherwise specified, a *p*-value cutoff of 0.01 after a Benjamini–Hochberg adjustment was considered significant across differential CpG or DMR statistical models. For M-value differences across comparison, normality was assessed through a qqplot and a histogram; after normality was determined (Figure S10), a paired *t*-test followed by a Bonferroni adjustment was conducted for M-value differences for each identified DMR for all contrasts; * *p*.adj < 0.05, ** *p*.adj < 0.01; *** *p*.adj < 1 × 10^−3^; **** *p*.adj < 1 × 10^−4^ showed significance.

### 2.12. ATP Assay

ATPlite ATP Luminescence Assay System (Revvity 6016736, Shelton, CT, USA) is a sensitive method to measure cell viability, proliferation, or cytotoxicity by detecting ATP concentration intracellularly, the cell’s energy indicator, using firefly luciferase. The analysis was conducted using manufacturer’s instructions.

### 2.13. Hypertrophy Assay

C2C12 cells were seeded into a 24-well flask. Cells were cultured in DMEM (Gen Clone MS014S) medium with 10% FBS. Four replicates were performed per experimental group using the control (OspA-ApoAI-CPP), IGF-1, or iDRIVE-GPR56 (OspA-ApoAI-GPR56-CPP). Myotube differentiation was performed after the cells reached 90–100% confluence using 2% Horse serum DMEM for four days [32]. Differentiated C2C12 myotubes were either treated with 100 ng/mL IGF-1 (Sino Biological 10598-HNAE, Sino Biological, Houston TX, USA) for 72 h, 1 µM iDRIVE-GPR56 every 24 h for 72 h, or left untreated (control). The cells were then fixed using 4% PFA for 10 min; then incubated with permeabilization buffer (PBS, 0.1% Triton X-100 (*v*/*v*) and 1% BSA) for 5 min; and incubated at 4 °C overnight with primary Anti-Myosin, 1:50 dilution (Developmental Studies MF20). Alexa Fluor 488 goat anti-mouse IgG was used as secondary antibody, 1:100 dilution (Invitrogen #A11059) in PBS and was incubated for 1 h in the dark. The cells were then stained with 2.5 mg/mL DAPI solution in 5 mL of PBS. Before imaging, 3 drops of Fluormount G were added to each well. Imaging was done in a Revolve ECHO microscope at 10× magnification. Each well was split into 4 pictures. Myotubes were measured 5 times through their diameter and averaged to produce one myotube diameter length. We performed at least 150 measurements per image, leading to 30 myotubes measured per image. In total, we measured 120 myotubes per well across four replicates per experimental group, i.e., the negative control, IGF-1, and iDRIVE-GPR56 treatments. Statistical analyses were performed using a pairwise Welch’s *t*-test on log transformed diameter lengths. Data normality was assessed visually through a qqplot and a histogram (Figure S10).

## 3. Results

### 3.1. Design of iDRIVE (In Vivo Deployment of Recombinant Viable MPs)

When we first designed water-soluble membrane proteins (wsMPs), we hypothesized that these would be tools in areas like structural biology. However, our first MP solubilization technique, SIMPLEx, was shown to retain not only the structurally relevant MPs’ conformations but also their functional integrity [17,19]. In our present work, our hypothesis is in part based on working with SIMPLEx and pore-forming proteins (PFPs), where SIMPLEx PFP seemed to be inserted into the plasma membrane of mammalian cells and negatively affect their growth in vitro (Section 3.2). Apolipoprotein AI (Apo AI) is one of the proteins employed in the SIMPLEx particles to solubilize MPs [17,19], and it is well documented to bind and solubilize lipids in lipoprotein particles (chylomicrons) and to interact directly with the plasma membrane to extract phospholipids and cholesterol [33]. Therefore, our hypothesis is further modified to include the following statement: reorganizing the triple fusion of proteins from SIMPLEx will result in a wsMP that can be enticed to interact with the plasma membrane by including a cell-penetrating peptide (CPP). We note that the sequence of the triple fusion is constrained by the presence of an MP. In 2015, Mizrachi and colleagues [17] demonstrated the need for an N-terminal decoy protein that allows nascent MP peptides to be folded and stabilized, via the fused Apo-AI, in the cytosol instead of being translocated to the hosts’ plasma membrane. Thus, the only possible permutation is as follows: decoy protein (OspA), Apo-AI, MP, and finally CPP.

**Figure 1:**
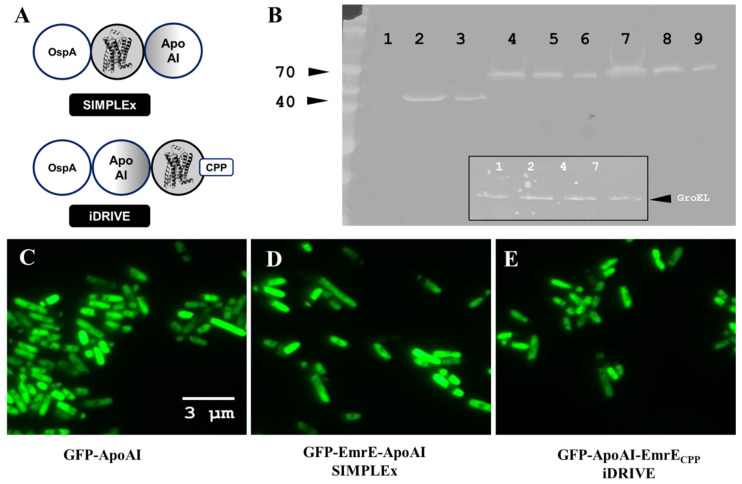
Strategic design of iDRIVE. (**A**) Graphical representation of the SIMPLEx or iDRIVE water solubilization of membrane proteins strategies. iDRIVE is a different permutation of SIMPLEx [35] with an added C-terminal cell-penetrating peptide (CPP). (**B**) Solubility of iDRIVE and SIMPLEx -EmrE. Western blot, anti-GFP. Soluble extracts of cells (BL21 DE3) expressing the different proteins tested. Lane 1 (negative control, un-transfected cells), Lanes 2–3 (GFP-ApoAI), Lanes 4–6 (GFP-EmrE-ApoAI), Lanes 7–9 (GFP-ApoAI-EmrE-CPP). Inserted into the main Western blot is a loading control using anti-GroEL for samples 1, 2, 4 and 7. (**C**–**E**) Confocal microscopy of BL21 DE3 cells expressing the proteins tested. Original figures can be found in Supplementary Materials.

The SIMPLEx MP water solubilization method uses a decoy soluble protein fused to the N-terminus of the membrane protein and Apo AI fused to the C-terminal of the membrane protein (Figure 1B, top panel). We propose a re-arrangement of the components of the SIMPLEx triple fusion (Figure 1B, bottom panel): the decoy remains as the most N-terminal position, followed by Apo-AI and the membrane protein fused at the C-terminal. We tested this rearranged triple fusion on EmrE, as in the 2015 Nat Comm article [17], and found that our new construct resulted in soluble membrane proteins, similar to SIMPLEx (Figure 1D,E). The final modification in our new strategy (iDRIVE) comes in the form of a CPP [34] that is placed on the C-terminal of the membrane protein. As demonstrated by Western blot (Figure 1C) or confocal images of *E. coli* hosting GFP-Apo AI or SIMPLEx-EmrE or iDRIVE-EmrE, our new design can be described as an alternative method to create wsMPs. We employed EmrE as our MP of choice since data already exist in the literature for its solubilization and, thus, creates a strong argument for our data evaluation.

### 3.2. SIMPLEx and iDRIVE Pore-Forming Proteins Affect Cell Membranes

**Figure 2:**
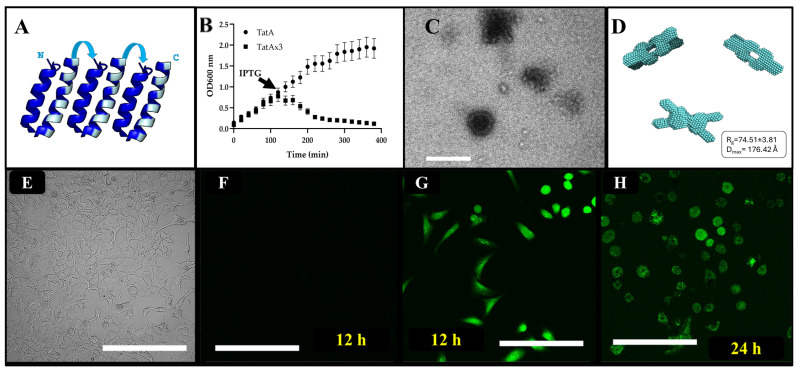
iDRIVE TATAx3, PFP. (**A**) Final design of the synthetic PFP. Based on the article by Walther 2013 [36], we hypothesized that the design of a synthetic PFP can be achieved through the “charge zipper” matching the transmembrane domain and the APH domain. Letters N and C correspond to the protein’s termini. (**B**) TATAx3 overexpression in BL21 DE3, directed by IPTG, resulted in decreased cell growth, measured at OD_600nm_ over time. Data collected from 5 independent experiments, each point in triplicate. Data is Average ± SD. (**C**) Negative staining image of SIMPLEx TATAx3, purified from BL21 DE3 and subject to uranyl acetate negative staining. As the protein forms pores, the uranyl acetate accumulates inside the pore. Scale bar corresponds to 500 nm. (**D**) Three different volume orientations of MBP-TATAx3-ApoAI predicted with small-angle X-ray scattering (SAXS). A pore is observed as the soluble protein appears composed of a homo-tetramer MBP-TATAx3-ApoAI. (**E**,**F**) HEK 293 cells after 12 h of exposure to GFP-ApoAI-CPP. (**E**) Light microscopy generated during the confocal microscopy. (**F**–**H**) Confocal microscopy of HEK 293 cells exposed to GFP-Apo AI-TATAx3-CPP; (**G**) after 12 h; or (**H**) after 24 h. (**E**–**H**) Scale bars correspond to 100 µm.

Initially, we engineered a synthetic pore-forming protein (PFP) based on the bacterial pore-forming Tat-A protein (https://www.uniprot.org/uniprotkb/P69428/entry, accessed on 30 January 2026) that has been proposed to fold during the pore-forming process which mediates membrane translocation [36]. Electrostatic “charge zippers” could be responsible for the transition of Tat-A from a single-membrane protein to a multimeric unit that creates the pore. Approximately one third of proteins synthesized in the bacterial cytoplasm pass the cytoplasmic membrane using a transport system that involves pore formation and signal peptides to direct protein export. In bacteria, the Twin-arginine translocation (TAT) pathway is more exclusive, with only 30 native substrates in the Gram-negative bacterium *E. coli*, and it is not universally conserved [37]. Each subunit of Tat-A consists of a transmembrane segment, an amphipathic helix (APH), and a C-terminal populated with charged amino acids (CAA) [38]. The sequence of charges in the transmembrane is complementary to the charges present on the APH, suggesting that the protein can be “zipped up” by as many as seven salt bridges [36]. The length of the resulting hairpin matches the lipid bilayer height; hence, a transmembrane pore could self-assemble via intra- and intermolecular interactions [36]. These steric interactions were characterized by molecular dynamics simulations by the authors [36]. Our team subscribed to this hypothesis and followed up using protein engineering to create a synthetic construct that reconstituted a truncated Tat-A in tandem by fusing three monomeric Tat-A helices between amino acids 1 and 48, which excludes the CAA domain (Figure 2B). We called this helical trimer subunit TATAx3 and expected it to be expressed as a membrane protein that might mediate its translocation into the membrane.

We then proceeded to generate water-soluble TATAx3 through the SIMPLEx triple fusion strategy (see Figure 2A). Moreover, we hypothesized that if TATAx3 inserts into the membrane, it might not harm the cells without further pore assembly. Interestingly, when bacterial cells express TATAx3 as a membrane protein under the direction of IPTG, cell number and proliferation decline until the population cannot overcome the process and disappears (Figure 2B). These data suggest that TATAx3 may be assembling beyond its single unit and may be recreating a pore, causing cellular death, either through irreparable membrane rupture or by triggering regulated apoptosis once pores disturb ionic and metabolic homeostasis [39]. In the literature [38], Tat-A has been reported to have an inner diameter of about 40 Å for a pore measured using electron microscopy at low resolution, which may correspond to 12–14 subunits. So, to further explore the pore-forming capabilities of SIMPLEx-TATAx3, we also examined it using negative staining (electron microscopy) and confirmed that under the conditions of expression and purification, the complex formed a pore with an outside diameter of 75 ± 7 nm and an inside diameter of 62 ± 7 nm (Figure 2C). These data seem to indicate that TATAx3 may assemble as a tetramer or pentamer. To validate this hypothesis, we opted for a structural approach. SIMPLEx-TATAx3 was the result of OspA-TATAx3-Apo AI fusion. Therefore, to create a bulging decoy protein that may provide more evidence of the assembly and number of subunits, we replaced OspA, a small globular protein, with maltose-binding protein (MBP) and analyzed it using small angle X-ray scattering (SAXS). The goal was to create a structural feature that could allow us to determine a definitive number of subunits in the pore-forming structure of TATAx3. Our results from SAXS using purified MBP-TATAx3-ApoAI validated that TATA-x3 formed a homotetramer in solution (Figure 2D).

Next, we conjectured that rearranging the SIMPLEx triple fusion to include a cell-penetrating peptide (CPP) at the C-terminus of the IMP would further mediate cell membrane interactions. So, given the need for an N-terminal small soluble decoy protein in the SIMPLEx fusion [17], we tested the only available permutation, GFP-ApoAI-TATAx3-CPP, and deployed our construct to HEK 293 cells in culture. Here, we replaced OspA with GFP to enable fluorescence monitoring of the experimental outcome. We hypothesized that the insertion of the pre-formed pores, TATAx3, would drive changes to the cell’s morphology and perhaps be sufficient to cause cell death [39], similar to our previous observations with the induction of TATAx3 expression in bacterial cells (Figure 2B). We tested HEK 293 cells at a density of 60% confluency in the presence of 2 µM of control GFP-ApoAI-CPP or iDRIVE-TATAx3 (GFP-ApoAI-TATAx3-CPP). As expected, the control protein (GFP-ApoAI-CPP) did not increase the level of observed fluorescence nor did it change the morphology of cells, implying that our control (GFP-ApoAI-CPP) did not insert into the plasma membrane nor was toxic to the cells (Figure 2E,F). In contrast, iDRIVE-TATAx3 (GFP-ApoAI-TATAx3-CPP) emitted sufficient green fluorescence even 12 h post treatment, as imaged through confocal microscopy (Figure 2G). Furthermore, 24 h after iDRIVE-TATAx3 treatment, HEK 293 cells depicted a round morphology, a sign of cellular stress and a well-recognized early morphological hallmark of apoptosis, as would be expected with inserted TATAx3 pores [40] (Figure 2H). In addition, cellular death or a strong disruption in the cell cycle may also be inferred since mammalian cells’ populations tend to double in a period of 24 h, and iDRIVE-TATAx3-treated cells did not seem to increase in confluency. Finally, puncta of fluorescent objects were observed in the interior of some of the cells, which may indicate the formation of condensed structures such as autophagosomes or stress granules in mammalian cells [41] (Figure 2H). Puncta from a GFP-fusion and cell rounding most often indicate protein aggregation/phase separation or stress responses (e.g., autophagy and ER stress, toxicity) [42,43]. Additionally, GFP foci/puncta, cell rounding, and osmotic shock are all linked phenomena under hyperosmotic stress. Our observations in Figure 2H could indicate osmotic-stress response rather than constitutive “true” puncta [44]. Further research is needed to validate this conjecture.

### 3.3. iDRIVE Interacts with Quiescent and Proliferating Cells In Vivo

**Figure 3:**
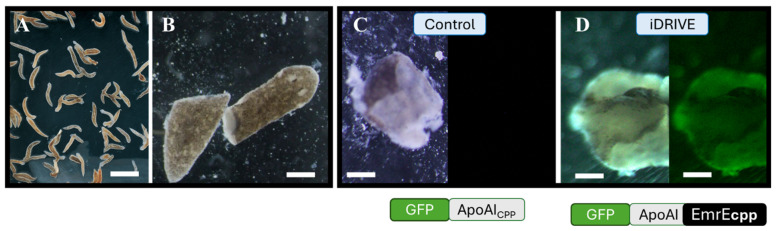
Planaria interactions with iDRIVE. (**A**) Planaria, typically visible with the naked eye. The scale bar corresponds to 1 mm. (**B**) Planarian that was transversally cut. Scale bar corresponds to 100 µm. (**C**,**D**) Light (Left) and confocal (right) microscopy imaging of planaria fragments treated for 48 h with (**C**) 1 µM GFP-Apo AI, with fluorescence not detected after fresh water was replaced, or (**D**) 1 µM GFP-Apo AI-EmrE-CPP (iDRIVE), with fluorescence detected throughout planaria’s periphery after fresh water was replaced. Scale bar corresponds to 100 µm.

We next sought to qualitatively test whether iDRIVE deployed to cells in vivo had any preferential interaction with cells that are more vulnerable or actively dividing. We used the planaria regeneration model, as they have a population of adult somatic stem cells, called neoblasts, that support continuous tissue turnover and whole-body regeneration, including individually pluripotent clonogenic cells [45,46,47]. Similarly to previous experiments (Figure 1D,E), we replaced the N-terminal decoy OspA in our iDRIVE construct with GFP to monitor the experimental outcomes. We then treated planaria that were regenerating a tail or a head for 48 h with either 1 µM iDRIVE_GFP_-EmrE or 1 µM GFP-ApoAI (control) [46]. Subsequently, we examined the treated fragments through fluorescent imaging. Not surprisingly, we did not detect fluorescence on fragments treated with our control construct (Figure 3C), which suggested that GFP-ApoAI is not sufficient to incorporate into the plasma membrane of quiescent and proliferating cells. In contrast, fragments treated with iDRIVE_GFP_-EmrE emitted green fluorescence that appeared brighter around the periphery of the organism, as would be expected for a membrane-inserting construct. Although the initial thought was to provide actively dividing cells with iDRIVE_GFP_-EmrE, i.e., the cells exposed after the cut, we detected fluorescence in many different structures of the planaria fragment. Thus far, iDRIVE seems to apply to both quiescent and proliferating cells.

### 3.4. iDRIVE Allows MPs to Re-Insert into the Plasma Membrane: Planar Bilayer Interactions

**Figure 4:**
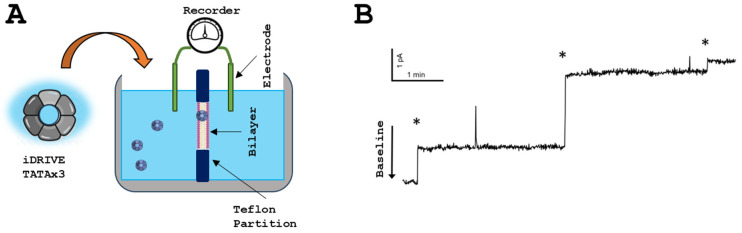
iDRIVE TATAx3 re-inserts into membranes. (**A**) Schematic representation of planar lipid bilayer (PLB) and experimental approach. (**B**) PLB recordings after one of the cis chambers is exposed to iDRIVE TATAx3. The asterisk (*) represents a TATAx3 insertion event.

**Figure 5:**
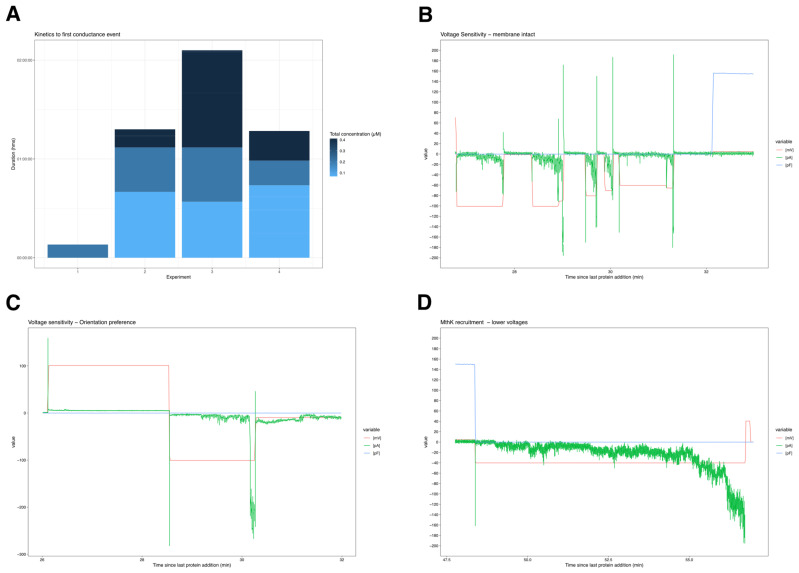
PLB experiments using iDRIVE MthK channel. (**A**) Protein incubation time required for first conductance event. (**B**) Voltage-dependent activation of iDRIVE MthK. (**C**) iDRIVE-MthK inserted in one orientation. (**D**) Recruitment of MthK channel opening, where “square-like” currents may represent individual channel opening.

Following our experiments that suggested that iDRIVE was mediating the insertion of wsMPs into cell membranes (Section 3.2), we sought to functionally assess these insertions. To this end, we performed planar bilayer electrophysiology using iDRIVE on either our pore-forming protein, TATAx3 (Figure 4), or MthK (Figure 5), a K^+^ channel from the archaean Methanobacterium thermoautotrophicum [27]. Our hypothesis was as follows: if iDRIVE mediates the insertion of “channel” membrane proteins and allows channel complex formation, we will be able to measure ion conductance changes in response to applied potentials across a planar lipid bilayer (PLB) [26]. Adding iDRIVE-TATAx3 to the cis chamber resulted in a “stepwise” conductance that reached equilibration in response to applied potentials (Figure 4). These “stepwise” conductance increases may suggest insertion events of the constitutively formed TATAx3 pore complex (Section 3.2).

To further characterize iDRIVE membrane insertions, we performed PLB experiments using the voltage- and calcium-sensitive tetrameric K^+^ channel, MthK [48] (Section 2.10). For these experiments, the cis chamber electrode was considered ground. The series of alternating voltages (100 mV or −100 mV) applied across the chambers before protein addition resulted in no conductance other than noise (abs.value < 10 pA), or capacitor current (Figure S5). In most experiments (*n* = 4), MthK concentrations of 0.07 µM with a mean duration of ~39 min, followed by 0.21 µM for a mean of 21 min, did not result in conduction events, except one where a conduction event was observed with 0.21 µM after 7 min of iDRIVE-MthK incubation. Rather, the first conductance event was recorded after an average of 29 min of additional incubation using a total of 0.4 µM iDRIVE-MthK (Figure 5A). We note that the addition of 1 mM of calcium in the cis and trans chambers did not produce a conductance event even after 59 min of incubation with 0.4 µM of iDRIVE-MthK. We speculate Ca^2+^ in the cis chamber may have caused a conformational change in the octameric C-terminal RCK domains, as previously reported [48,49], and this may have interfered with the protein membrane insertion, as the first conductance event was immediately triggered after the addition of 2 mM EDTA in both the cis and trans chambers (Figure 5A and Figure S4). Further studies are needed to validate this speculation.

Once conductance events were established, we observed that conductance showed a voltage-dependent response, favoring highly negative voltages and silencing with increased depolarization (positive voltages). Indeed, negative voltage were observed to “rescue” ion conductance, whereas positive potentials silenced ion conductance, as would be expected with inserted MthK channels (Figure 5B,C), consistent with the open channel probabilities observed by Thomson and Rothberg in 2010 [50] and Posson et al. in 2013 [51]. This voltage preference also suggested that the observed conductance was not due to any bilayer leakages, as ionic flow is irrespective of potential polarities. Furthermore, given the high open probability at high negative voltages (−100 mV), we tested voltages that correspond to lower open probability, as previously reported, and observed a gradual recruitment-like behavior where there seemed to be individual channel activations adding over time and yielding a geometric or exponential increase in conductance, as opposed to the drastic conductance quickly triggered by more negative potentials (Figure 5D).

Our observations also allow us to infer ion current directionality. First, given that MthK has been shown to exhibit a strong ionic preference for K^+^ and Rb^+^ [50] and each of its subunits’ selectivity filters (TVGYGD) is predicted to have a charge z~−1.9 at the tested pH 8.02, the measured negative conductance highly likely indicated a cis (ground) to trans flow of cations, and we rule out an anion interpretation (Figure S7). Additionally, seeing that MthK is an inward rectifier K^+^ channel, i.e., it favors inward ion conductance and has higher open probabilities at negative voltages and much lower at positive ones [51], we can infer that iDRIVE is mediating the protein insertion of MthK in one orientation. This is evidenced by the stark conductance differences between voltages of the same magnitude but of different polarities, more specifically, the lack of ionic conductance at positive potentials (Figure 5C and Figure S6). If iDRIVE-MthK was inserting in multiple orientations, we would expect conductance at both positive and negative potentials. This is not what we observed. Therefore, we can infer that the cis (ground) chamber houses the extracellular N-terminus of iDRIVE-MthK, and negative currents show “inward” cation flow from the cis (extracellular) to the trans (intracellular) chambers. This interpretation is also the simplest one, as the cis chamber (extracellular space) would house the bulkiest water-soluble domain of iDRIVE-MthK, OspA, and ApoAI (34.52 kDa), as it would seem less likely that these water-soluble domains of the fusion protein are enticed to cross over the lipid bilayer and orient themselves in a trans (extracellular) to cis (intracellular) fashion. If that were the case, the voltage with which conductance was favored would correspond to a positive voltage, but 1. MthK’s open probability decreases drastically with positive potentials; 2. our observed negative current would correspond to cations moving from the intracellular to the extracellular space, but MthK is inward rectifying; and 3. this conformation would require the bulkier water-soluble region (OspA-ApoAI) to cross over the bilayer. Consequently, we conclude that iDRIVE mediates the membrane insertion in an orientation specific manner, with its C-terminus being enticed to the membrane and its N-terminus staying in the extracellular side. Following Occam’s razor, we remark that this interpretation is consistent with the cis chamber (extracellular space) 1. housing the bulkiest water-soluble domain of iDRIVE-MthK, i.e., OspA and ApoAI (34.52 kDa); 2. aligning voltage-dependent polarity with open probabilities reported for MthK, i.e., higher negative voltages result in higher conductance; and 3. corresponding negative conductance with “inward” cation currents, consistent with the inward MthK rectification property. Of interest, we note that MthK has two water-soluble C-terminal RCK domains [27] with weights of 12.98 and 9.26 kDa. Notwithstanding, our results showed that neither our construct nor these water-soluble RCK domains prevented the iDRIVE-mediated MthK membrane bilayer insertion or MthK channel complex assembly and functionality.

### 3.5. iDRIVE Allows MPs to Activate Signal Pathways In Vitro: The Case of Myotube Hypertrophy

**Figure 6:**
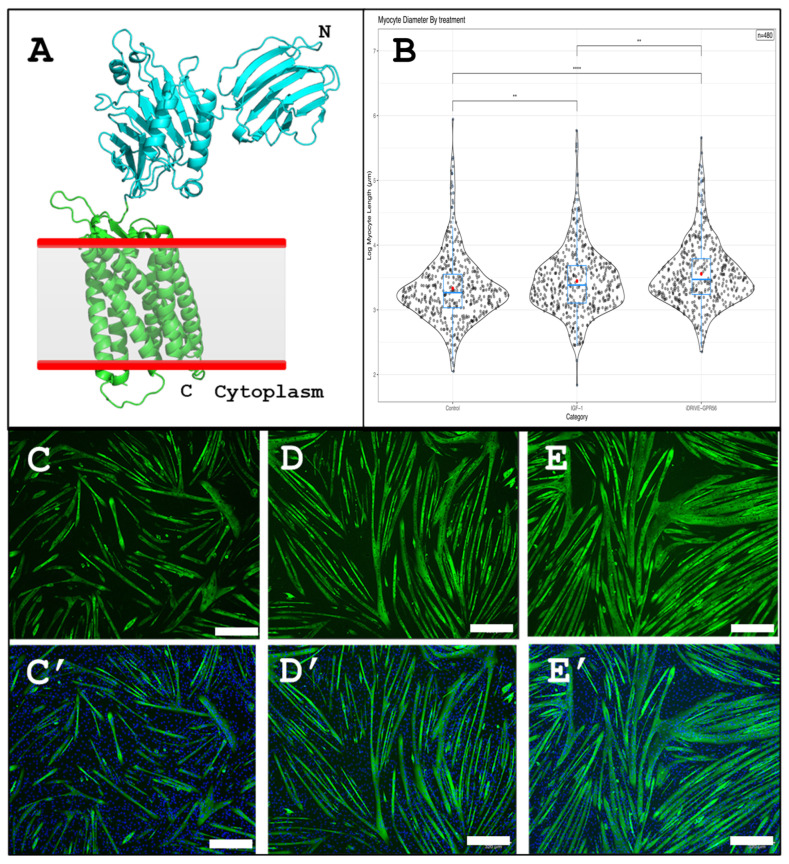
Myotube hypertrophy assay. (**A**) Representation of an adhesion GPCR (aGPCR). The N-terminus (light blue) is extracellular and is responsible for establishing contact and adhesion to other cells. (**B**) Sina plot of myotube diameter measurements performed when C2C12 cells are left untreated (control) or are exposed to 10 ng/mL IGF-1 or 1 µM iDRIVE-caGPR56. Statistical significance between groups is represented by ** *p*.adj < 0.01; **** *p*.adj < 1 × 10^−4^. (**C**,**C′**) C2C12 cells, no-treatment. Green corresponds to Calcein, and blue is DAPI staining. (**D**,**D′**) C2C12 cells, IGF-1 treatment. (**E**,**E′**) C2C12 cells, iDRIVE (GPR56) treatment. Scale bars correspond to 300 µm.

Encouraged by our previous results, we set out to test if iDRIVE can deliver active signaling molecules to the cellular plasma membrane and alter cellular fate. We assessed muscle fiber hypertrophy in murine C2C12 cells. In classical experiments, C2C12 cells are treated with insulin growth factor-1 (IGF-1) to induce myotube hypertrophy [52]. Of interest, adhesion G-protein coupled receptors (aGPCRs) like GPR56 have also been associated with myotube hypertrophy [53]. We proposed then to design a constitutively active GPR56 (caGPR56) iDRIVE construct and compare it to IGF-1 in a myotube hypertrophy assay in murine C2C12 cells. Previously, it has been shown that it is possible to generate a constitutively active GPR56 (caGPR56) to induce myotubule hypertrophy [54]. Constitutive activity is the ability of a GPCR to undergo agonist-independent isomerization from an inactive (R) state to an active (R*) state [55]. More specifically, the family of aGPCRs has a large extracellular domain that is typically connected with the surface of a neighboring cell [56] (Figure 6A); they also contain an autoproteolysis-inducing (GAIN) domain, a unique and essential protein domain found in most aGPCRs that mediate their activation and self-cleavage into two fragments: an N-terminal fragment (NTF) and a C-terminal fragment (CTF) [56]. The GAIN domain includes a tethered agonist sequence called the Stachel, which is crucial for activating the receptor [56]. While many aGPCRs undergo this process, some are activated without cleavage or via other domains, like the SEA domain in GPR110 [57]. We removed the extracellular domain of GPR56 to produce its constitutively active version (caGPR56) [56,57].

We divided our experimental groups into untreated (control), IGF-1-treated, or iDRIVE-caGPR56 treatment. Promisingly, we found that IGF-1 and iDRIVE-caGPR56 significantly increased the myofiber major axis diameter of C2C12 relative to untreated fibers. We also observed that iDRIVE-caGPR56 treatment resulted in significantly increased mean fiber diameters relative to IGF-1 treatment (Figure 6B); however, this may have been due to the higher frequency with which fibers were treated relative to IGF-1 (Section 2.12). However, the fiber hypertrophy phenotypic convergence is interesting, given that IGF-1 [58] and GPR56 [56] rely on signaling pathways that use very different signaling modalities, i.e., chemical vs. mechanical, respectively. This result is consistent with Dr. Michael Levin’s (Department of Biology, director of the Allen Discovery Center at Tufts University; https://drmichaellevin.org/, accessed on 26 March 2026) morphospace hypothesis, where biological systems may converge in anatomical and morphological configurations, via differently activated pathways [59,60]. In our case, we showed that in vitro hypertrophy can be reached via IGF-1 or iDRIVE-caGPR56. And although, in the literature, IGF-1 is related to the mechanically signaling gap junctions [61], to the best of our knowledge, there are no reported cross signaling interactions between IGF-1 and the adhesion protein GPR56 [62,63].

### 3.6. iDRIVE Allows MPs to Activate Signal Pathways In Vitro: The Case of Embryoid Body Differentiation

Having observed that iDRIVE was successful in solubilizing MPs and mediating their incorporation into plasma membranes and effecting a functional change to cells in culture (Figure 6), we sought to solidify the evidence by testing one more system in vitro. We tested iDRIVE in the context of directing HEK 293 embryoid body (EB) differentiation through the Wnt signaling pathway. Wnt signaling is critical for proper embryonic development and for tissue homeostasis in adults [64]. Activation of this signaling cascade is initiated by binding the secreted Wnt to their receptors, Frizzled (FZD) [65]. Wnt signaling transduction relies on the activation of β-catenin, which is normally sequestered by a protein complex that prevents its migration to the nucleus, thereby inhibiting Wnt-mediated downstream gene expression changes [66]. In the laboratory, a small molecule, CHIR99021, can be used to stimulate the Wnt/β-catenin pathway [67]. CHIR99021 inhibits GSK-3α and GSK-3β at low-nanomolar IC_50_ values [68]. Namely, CHIR99021 blocks GSK-3–dependent phosphorylation of β-catenin, preventing its ubiquitination and proteasomal degradation; this matches the main mechanism of action of Wnt/FZD downstream activity [65,66,68]. To evaluate this system with iDRIVE, we designed a constitutively active human Frizzled 7 receptor (cahFZD7), following the strategy published by Xu and colleagues in 2021 [69], to activate signal transduction mediated by the Wnt/β-catenin pathway in the absence of Wnt.

Embryoid spheroids (ESs) are a simple form of 3D cell structures that improve the in vitro recapitulation of native cellular behavior, relative to 2D cultures [70]. So, we generated HEK 293 ESs, as previously described (Figure S2) [70], and set out to investigate the potential for iDRIVE to be used in the spheroid differentiation. We used HEK 293 cells, as they are a standard model to study Wnt-FZD interactions and activation of the canonical Wnt/β-catenin pathway [71], as they naturally express various Frizzled (FZD) receptors (e.g., FZD2, FZD7, FZD1, FZD4, and FZD9) and Wnt signaling components. More specifically, ESs provided an opportunity to monitor the diffusion of iDRIVE into the inner cells of the EB mass and direct cellular fate. This strategy was used in preparation to test iDRIVE in vivo. We hypothesized that with differentiation, energy demand would increase. And, as ATP is produced and further hydrolyzed to ADP/AMP, it will stimulate key metabolic enzymes and pathways that further ramp up ATP production [72]. Consequently, we measured the ATP levels of our differentiating ESs across treatments (Figure S2). We observed that neither CHIR99021 nor iDRIVE-cahFZD7 increased ATP levels by themselves (Figure S2). As such, following a recent report [73] that the combination of CHIR99021 and Retinoic acid (RA) was successful in differentiating human embryonic stem cells (hESCs) into primordial germ cells (PGCs) and that this process has been shown to modulate the Wnt ligand/receptor expression in a cell-dependent manner [72,73], we decided to test RA (10 µM) in our ESs in conjunction with CHIR99021 or with iDRIVE using a FZD receptor. We chose human FZD7, to create, based on the literature, a constitutively active human FZD7 [69], iDRIVE-cahFZD7. This will afford us the ability to deliver an active Wnt signaling independent of Wnt. In contrast to our previous results, we observed that when CHIR99021 or iDRIVE-cahFDZ7 were combined with RA, there was a significant increase in measured ATP levels in treatment groups relative to the treatment with RA alone or untreated cells (Figure S2). These results highlighted the sufficiency for either iDRIVE-cahFDZ7 or CHIR99021, together with RA, to recapitulate the bioenergetic improvements observed in our in vitro ESs. We propose that the observed increase in bioenergetic demands from both iDRIVE-cahFDZ7 or CHIR99021 and RA were due to Wnt-mediated activity. Moreover, we hypothesize that the higher ATP demand measured in RA+iDRIVE-cahFZD7 was due to a more thorough Wnt signaling activation, as opposed to that of RA+CHIR99021 (Section 3.8).

### 3.7. iDRIVE Allows MPs to Activate Signaling Pathways In Vivo: The Case of Planaria Regeneration

**Figure 7:**
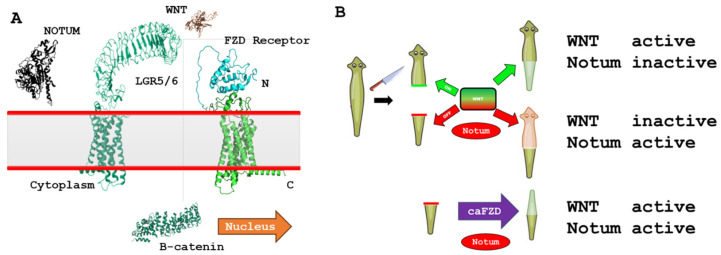
Basic elements of experimental design. (**A**) The important molecules in the Wnt signaling pathway. Important elements in Wnt signaling pathway in planaria regeneration. FZD (frizzled) receptors, LGR5 co-receptor, and β-catenin are the intracellular targets, and Notum inactivates Wnt signaling. (**B**) The strategy followed and the outcomes proposed upon treatment. Normal Wnt-mediated planarian head and tail regeneration contrasted with iDRIVE-capFZD treatment-mediated regeneration. Planarian tail regeneration relies on Wnt activation, with Notum (Wnt’s antagonist) inactive. Planarian head regeneration relies on Notum activation, which consequently, will inactivate Wnt. Notum activation in tails regenerating heads will influence native extracellular components of Wnt signaling, but not on the capFZD, enabling it to signal.

**Figure 8:**
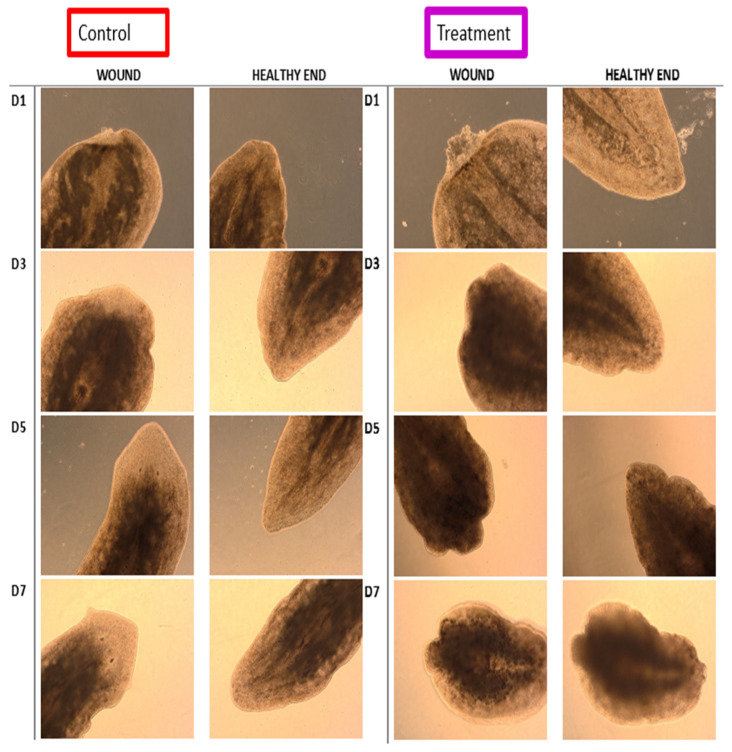
Planaria regeneration. Planaria tail regenerating head (Wnt OFF) time course. Treatments are divided into two groups, with images being separated for the wound and the tail of the same planarian, for clarity. Images are taken each day (D) after the surgery. Control (**Left**), tails treated with 0.5 µM iDRIVE-GFP (OspA-ApoAI-GFP-CPP), or constitutively active FZD (**right**) using 0.5 µM iDRIVE-capFZD. Picture sequence indicates time post transversal cut. Day 1 (D1), 24 h post wound, day 3 (D3), 72 h, etc. Day 8 pictures were also taken for the treatment group and showed no evidence of head regeneration.

Our final experiment aims to demonstrate that iDRIVE can trigger signaling transduction in vivo. We will use the Wnt/FZD/β-catenin signaling pathway as a tool to qualify the outcome of planaria regeneration (Figure 7). As previously reviewed, planaria are freshwater flatworms with remarkable regeneration of whole body parts—including brain, gut, and organs—from tiny fragments via pluripotent neoblasts [74]. When planaria are transversally cut, two wounds form. In spite of having a similar origin, these wounds will develop different programs that direct the regeneration of a tail or a head. In the head segment, the wound will follow Wnt signaling in the neoblasts to regenerate the tail [75]. In the tail, Wnt signaling will be initially active; nevertheless, cells will produce an enzyme called Notum that will inhibit Wnt signaling, and neoblasts will regenerate a head (Figure 7) [75]. Notum directly deacylates Wnt ligands, removing the essential palmitoleate group required for binding to Frizzled receptors, thereby inactivating Wnt proteins and suppressing downstream signaling [76]. Thus, the expression of Notum results in inactivation of Wnt signaling, and ultimately, leads to the regeneration of a head. In conclusion, Wnt ON results in a tail, and Wnt OFF results in a head (Figure 7). As an example, recent studies have generated two-tailed planaria by manipulating Wnt signaling using RNA interference (RNAi) [62,64] or altering bioelectric patterns in regenerating planaria [77,78,79]. Success in altering planaria regeneration has never been achieved using proteins or wsMPs. Following husbandry protocols, we prepared planaria for the experiment. After transversal cuts, tails were separated and exposed to 0.5 µM of constitutively active planaria FZD7 (iDRIVE-capFZD) or 0.5 µM of control protein, iDRIVE-GFP (OspA-ApoAI-GFP-CPP). The constitutively active FZD was the translation of the mutations performed in human FZD7 [69]. We tested two planaria FZD receptors (pFrizzled4mSx2 and pFrizzled1/2/7mSx2), with similar results. The data presented in this manuscript corresponds to pFrizzled1/2/7mSx2 and will be referred to as capFZD. The process of full-head regeneration was achieved between 5 and 7 days for control treatments (Figure 8). In contrast, we observed that treated tails (iDRIVE-capFZD) did not regenerate a head, as expected. This “two-tailed” phenotype presented in approximately 30% of the treatments, under the direction of iDRIVE-capFZD (*n* = 9 experiments).

**Figure 9:**
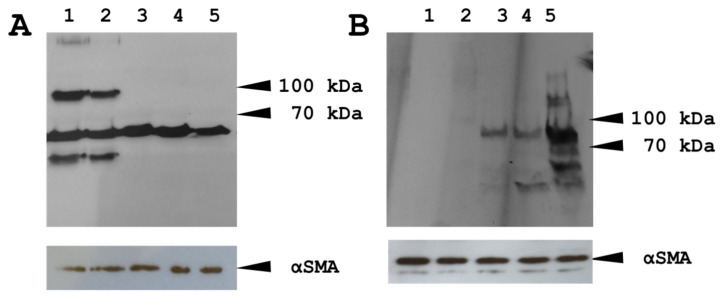
Analysis of planarian regeneration. (**A**) Western blot of planaria wounds 30 h post cut. Antibody (phospho-β-catenin). As a load control, we used α-smooth actin. Samples 1 and 2 correspond to untreated tails; samples 3 and 4 correspond to tails treated with iDRIVE-capFZD; and sample 5 corresponds to heads regenerating their tail. (**B**) Western blot of planaria wounds 30 h post cut. Antibody (β-catenin). As a load control, we used α-smooth actin (αSMA). Samples 1 and 2 correspond to untreated tails; samples 3 and 4 correspond to tails treated with iDRIVE-capFZD; and sample 5 corresponds to heads regenerating their tail (positive control). Beta catenin appears at ~92–95 kDa on Western blots. Phosphorylated forms migrate at the same apparent molecular weight (~92–95 kDa) due to minor mass shifts from phosphorylation that are typically undetectable on standard SDS-PAGE. Original figures can be found in Supplementary Materials.

To further elucidate the activity of the Wnt signaling pathway in the observed headless planaria phenotype, we performed Western blots of Beta-catenin (Figure 9B) or phosphorylated Beta-catenin (Figure 9A). Specifically, we assessed the following experimental design: tails regenerating heads treated with control (iDRIVE-GFP, Wnt OFF), heads regenerating tails with control (iDRIVE-GFP, Wnt ON), or tails regenerating tails treated with iDRIVE-capFZD (Wnt ON). As expected, control tails regenerating heads (Wnt OFF) exhibited expression of Phospho-Beta catenin but no catenin (Lanes 1, 2). Similarly, the positive control of heads regenerating tails (Wnt ON) showed Beta catenin expression but no Phospho-Beta catenin (Lane 5). In contrast, tails regenerating heads treated with iDRIVE-capFZD (Wnt ON) showed no Phospho-beta catenin but instead expressed the active beta catenin form (Lanes 3 and 4). Moreover, considering that Western blots are difficult to perform in planaria due to the lack of antibody specificity, we provide an alternative blot obtained with a different set of antibodies in the supplement (Figure S3). These results confirmed the activation of the Wnt signaling pathway on planarian tails regenerating heads and, thus, validated iDRIVE as a method to induce targeted signaling pathway changes and alter cell fate in vivo in a similar fashion as RNAi or bioelectricity manipulation in planarian regeneration.

The tool presented in this work, iDRIVE-capFZD, offers new paradigms to the study of cell regeneration, proliferation, and targeted signaling transduction and may offer new insights into its unique genetic and molecular downstream effects. More specifically, our tool may provide a new paradigm to study canonical Wnt vs. non-canonical Wnt signaling. Canonical Wnt signaling is mediated by Wnt–Frizzled (Fz)–LRP5/6 complexes, which inhibit β-catenin degradation and drive TCF/LEF transcription. Non-canonical pathways include Wnt/PCP (Fzd–disheveled–RhoA/JNK for polarity/migration) and Wnt/Calcium (Fzd–G proteins–PLC/IP3R–Calcium/NFAT for asymmetric cell division) [80]. The study of constitutively active receptors of the GPCR class has not advanced sufficiently to differentiate which pathway (canonical or non-canonical) may be activated [81]. We showed that our tool can present new avenues for research opportunities.

### 3.8. iDRIVE cahFZD7 and CHIR99021 Elicit Similar Differential Methylation Patterns Across CpG Regions—In Vitro HEK 293 Embryoid Bodies

**Figure 10:**
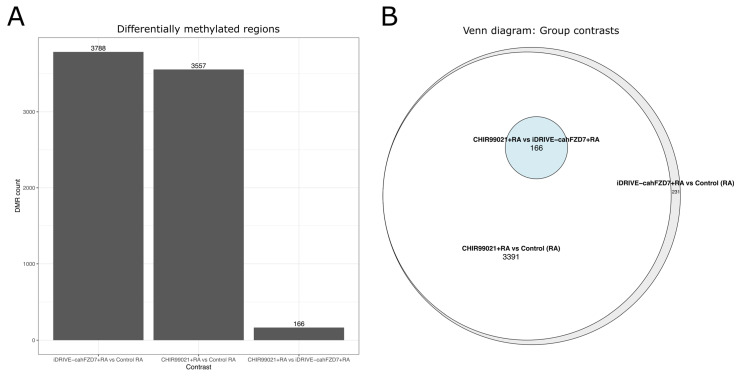
(**A**) Bar plot of significant DMRs across comparisons. (**B**) Venn diagram of significant DMRs across comparison. The 3788 DMRs found in the iDRIVE-cahFZD7+RA vs. control (RA) formed a superset of the 3557 DMRs in CHIR99021+RA vs. control (RA), which formed a superset of the 166 DMRs in CHIR99021+RA vs. iDRIVE-cahFZD7+RA.

**Figure 11:**
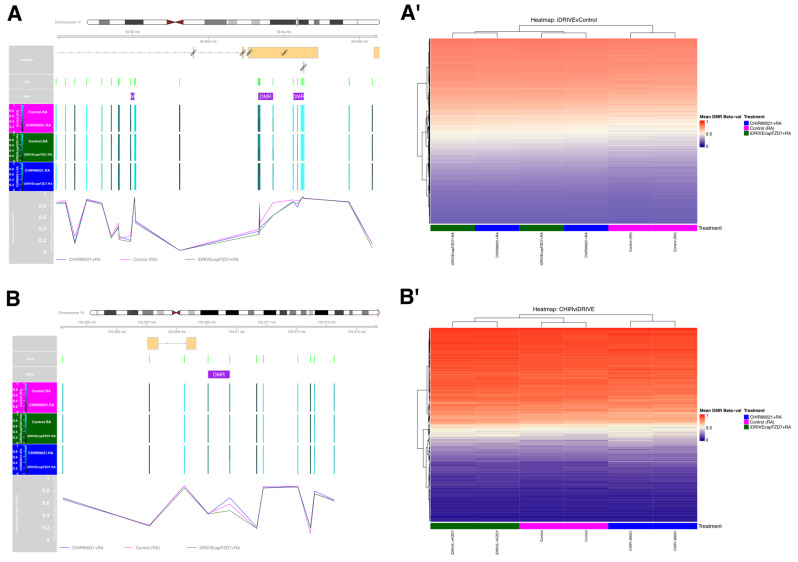
(**A**) DMR plot of region: Chr17 59838304-59839282. iDRIVE-cahFZD7+RA vs. control (RA) contrast. (**A′**) Heatmap of average Beta values across significant DMRs (3788) for iDRIVE-cahFZD7 + RA vs. control (RA) contrast. (**B**) DMR plot of region: Chr10 133069051-133069774. CHIR99021+RA vs. iDRIVE-cahFZD7 + RA contrast. (**B′**) Heatmap of average Beta values across significant DMRs (166) for CHIR99021 + RA vs. iDRIVE-cahFZD7 + RA contrast. Hierarchical clustering across samples shows iDRIVE-cahFZD7+RA to be more similar to control groups than to CHIR99021 + RA, for this set of DMRs.

Lastly, to further characterize the molecular effects of the Wnt signaling pathways induced by either CHIR990221 or constitutively active human FZD (iDRIVE-cahFZD7), we performed a differential methylation analysis on HEK 293 embryoid bodies treated with either CHIR990221+Retinoic Acid (RA), iDRIVE-cahFZD7+RA, or RA alone (control) (Section 3.6). All permutations of the available contrasts were assessed, and for simplicity and clarity, inclusion of RA and mention of control groups will be implicit when discussing contrasts, namely CHIR vs. control, iDRIVE vs. control, and CHIR vs. iDRIVE (Section 2.10). EPIC v2 array analyses resulted in 927,532 CpG loci that passed quality control across treatment groups. No individual CpG position was significantly different across comparison. However, we found 3788 differentially methylated regions (DMRs) with iDRIVE-cahFZD7 treatment, 3557 DMRs with CHIR99021, and 166 DMRs between CHIR99021 and iDRIVE-cahFZD7 (Figure 10A). Interestingly, the 3788 DMRs identified with iDRIVE-cahFZD7 treatment formed a superset of each of the other identified DMRs, demonstrating shared differential regions of methylation across comparison. More specifically, the aforementioned 166 DMRs were a subset of the 3557 DMRs, which in turn were a subset of the 3788 DMRs (Figure 10B). To further assess differential methylation patterns, significantly identified DMRs were compared for each experimental contrast. We observed that DMR patterns in iDRIVE-cahFZD7 closely followed DMR patterns found in CHIR99021 (Figure 11A,A′, Figure S11 and Figure S12). In contrast, DMRs between CHIR99021 and iDRIVE-cahFZD7 either showed iDRIVE-cahFZD7 following a unique methylation pattern that was different from the control and CHIR99021 treatment (Figure 11B and Figure S13) or a pattern more similar to the control, as seen by hierarchical clustering of average DMR Beta values across samples (Figure 11B′).

#### 3.8.1. Statistical Testing

**Figure 12:**
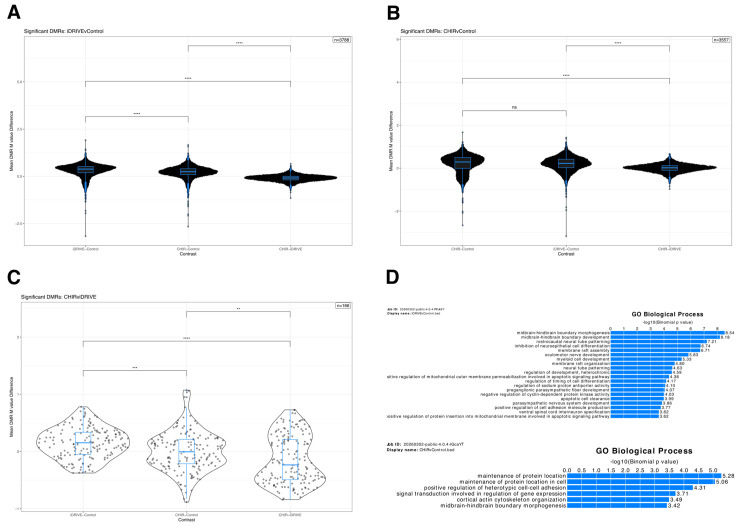
(**A**–**C**) Mean DMR M-value difference across contrasts. Significance tested through a paired *t*-test with Bonferroni *p*-val adjustment, ** *p*.adj < 0.01; *** *p*.adj < 1 × 10^−3^; **** *p*.adj < 1 × 10^−4^. (**A**) iDRIVE-cahFZD7+RA vs. control (RA). (**B**) CHIR99021+RA vs. control (RA). (**C**) CHIR99021+RA vs. iDRIVE-cahFZD7+RA. (**D**) DMR GO biological process enrichment—**top**: iDRIVE-cahFZD7+RA vs. control (RA)—**bottom**: CHIR99021+RA vs. control (RA).

Average DMR M-value differences were then compared across contrasts and used for significance testing. Average DMR M-value differences across contrasts showed that, for the DMRs identified with the CHIR99021 treatment, there was no statistical difference between CHIR99021 and iDRIVE-cahFZD7 groups against their control groups, suggesting that DMRs affected by CHIR99021 are also affected by iDRIVE-cahFZD7. Indeed, for this set of DMRs, the mean M-value difference between CHIR99021 and iDRIVE was ~0.007, which highlighted very similar DMR methylation patterns (Figure 12B). In contrast, when considering the DMRs identified in response to iDRIVE-cahFZD7, we saw a significant difference between all contrasts. This was interesting, because, as previously discussed, DMRs found with CHIR99021 treatment did not have significant average M-value differences with the same iDRIVE-treated DMRs. This suggests that for the superset of DMRs found with the iDRIVE-cahFZD7 treatment, CHIR99021 was similar to iDRIVE-cahFZD7, but the additional DMRs identified with iDRIVE-cahFZD7 treatment drove the significant differences between these two treatments. In fact, the mean M-value difference between CHIR99021 and iDRIVE-cahFZD7 for this set of DMRs was ~−0.09, which suggest that there are slightly more methylated DMRs with iDRIVE-cahFZD7 treatment than with CHIR99021 (Figure 12A). This notion is further supported by looking at only the set of significant DMRs in the CHIR99021 vs. iDRIVE-cahFZD7 comparison, where each contrast was also statistically different. Specifically, for this set (166 DMRs), iDRIVE-cahFZD7 vs. control had a mean M-value difference of ~0.15 whereas CHIR99021 and iDRIVE-cahFZD7 had a mean M-value difference of ~−0.14, reinforcing the notion that for the DMRs that are differently methylated between iDRIVE-cahFZD7 and CHIR99021, iDRIVE-cahFZD7 tended to result in more methylated regions than CHIR99021 (Figure 12C).

#### 3.8.2. Functional Enrichment

Lastly, functional enrichment of each of the DMRs across comparison was performed using the Stanford GREAT tool. iDRIVE-cahFZD7 treatment yielded the most enriched GO biological [82,83] processes with 20 enriched terms, followed by CHIR99021 with 6 enriched terms, whereas CHIR99021 vs. iDRIVE-cahFZD7 yielded no enrichments. Additionally, CHIR99021 and iDRIVE-cahFZD7 treatments had a common enrichment, namely, the “midbrain hindbrain morphogenesis” biological process. Interestingly, at least 11 of the 20 enriched iDRIVE-cahFZD7 GO biological processes involved developmental processes whereas CHIR99021 treatment resulted in at most two terms related to development. Similarly, GO molecular function enrichment resulted in three terms for iDRIVE-cahFZD7 and one term for CHIR99021 DMRs. GO cellular components were only enriched with CHIR99021 treatment, one term. Moreover, DMRs found using CHIR99021 or iDRIVE-cahFZD7 treatments had a similar number of associated genes, with iDRIVE-cahFZD7 tending to be associated with more genes than CHIR99021. Similar trends could be seen with DMRs’ distances to transcriptional start sites (TSSs) (Figure S14). In short, functional enrichment of DMRs across comparisons showed that iDRIVE-cahFZD7 not only recapitulates CHIR99021 downstream effects but may also even induce more biologically relevant and specific Wnt activation.

## 4. Discussion

Considering that proteins have a finite life span in the cell, iDRIVE signaling may result in phenotypic or genotypic driven changes, without the permanent sequelae of genetic manipulations, for example, transgenic animal models or viral infections. These manipulations can also replace “promiscuous” small molecules or toxic signals in the process of stem cell differentiation. Our strategy may also be extended to other GPCRs, which is the largest family of membrane protein receptors, ~800–1000 genes (~4–5% of the human genome) [84,85]. This is relevant since approximately 35% of FDA-approved drugs (~500–700 agents) target ~108–134 GPCRs, generating $180B+ annual sales, and underpin β-blockers, opioids, antihistamines, and antipsychotics, among many others [86], and if only ~15% of all human proteins are druggable and >60% of approved targets are membrane proteins, then the vast majority of membrane proteins—on the order of thousands—are still undrugged and, in practice, undruggable by current approaches. Using our method, we demonstrate that Wnt signaling pathway activation via the conventional small molecule agonist, CHIR99021, or our wsFrizzled (iDRIVE-FZD) receptors induced similar differential methylation patterns in human embryonic kidney (HEK 293) embryoid spheroids (ESs). Moreover, we showed that Wnt activation via wsFrizzled receptors resulted in even more biologically relevant methylation changes than via the small molecule CHIR99021. Nevertheless, we note that when using our method, authors should always consider the topology and functionality of their MP of interest, e.g., we note that iDRIVE-MthK would not display its known N-mediated channel inactivation [87] due to the nature of the fusion protein. Additionally, MP size may be another consideration. Larger targets will need special adjustments, particularly in the shield portion (Apo-AI) of the triple fusion. Previously, this was accomplished with further modifications of Apo-AI [19]. Of interest, a recent preprint by the laboratory of Dr. Matthew DeLisa (Professor in the School of Chemistry and Biomolecular Engineering, and Director of Cornell Institute of Biotechnology, Cornell University) indicated that further engineering can solve issues for proteins with up to 13 transmembrane helices [88]. Other limitations may be related to the composition of the plasma membrane of the target cells. Additionally, several well-defined GPCR subsets absolutely require a coreceptor, accessory protein, or chaperone for correct trafficking and/or signaling [89,90,91]. Nonetheless, with the proper experimental considerations, we are confident that our method offers a simple yet effective solution that provides a targeted access to functional MPs as an increment in solving the undruggable genome and that synthetic biology will continue to categorically provide solutions to “insoluble” problems [9].

## 5. Conclusions

We have presented evidence that our new protein engineering strategy, iDRIVE, solubilizes MPs. We also presented data that strongly suggest that iDRIVE mediates MPs’ re-insertion into the lipid bilayer of cells in vitro and in vivo. We also identified properties of the iDRIVE system that enable signaling MPs to actively drive instructions related to their native program, e.g., the use of constitutively active receptors. Furthermore, we suggest that wsMPs in the iDRIVE system can be used as tools to replace DNA/RNA manipulations of cells, tissues, or organisms. In our experiments comparing iDRIVE cahFZD7 and CHIR99021, the data seem to indicate they might be comparable when affecting β-catenin levels, with subsequent cell signaling. We also present data that seem to indicate iDRIVE may have further effects in cellular fate, as indicated by differential methylation analysis. CHIR99021 is a highly potent, selective aminopyrimidine inhibitor of Glycogen Synthase Kinase-3 (GSK-3α/β). It activates Wnt/β-catenin signaling and is widely used to maintain stem cell pluripotency, reprogram somatic cells to iPSCs, and drive differentiation. As such, we propose that iDRIVE cahFZD7 or other iDRIVE applications may be an alternative to small molecules in cell differentiation.

## Data Availability

The original contributions presented in this study are included in this article/its Supplementary Materials. Further inquiries can be directed to the corresponding author.

## Supplementary Materials

The following supporting information can be downloaded at: https://www.mdpi.com/article/10.3390/biom16040546/s1. Supplementary Table S1. Plasmids, accession number and amino acids sequence of constructs in this article. Figure S1. Epigenetics Sample metadata. Figure S2. iDRIVE-Frizzled + RA stimulate HEK 293 embryoid body bioenergetics. Figure S3. Phospho-β-catenin and β-catenin western blot. Figure S4. MthK incubation and free calcium concentration. Figure S5. Planar bilayer conductance quality control. Figure S6. iDRIVE-MthK re-inserts the lipid bilayer via its C-terminus. Figure S7. MthK selectivity filter Titration Curve. Figure S8. EPICv2 array sample and probe quality control. Figure S9. Comprehensive EPICv2 array methylation quality control. Figure S10. Normality assessments. Figure S11. Average DMR Beta values for significant DMRs in the CHIR99021+RA vs RA contrast. Figure S12. DMR region plots: iDRIVE-cahFZD7+RA vs Control (RA). Figure S13. DMR region plots: iDRIVE-cahFZD7+RA vs CHIR99021+RA. Figure S14. Associated gene count per DMR and absolute distance to TSS.
