# Supplementary material for: Beyond Small Molecules: Orchestrating Cell Fate with Engineered Water-Soluble Membrane Proteins
Source: Biomolecules. 2026 Apr 8;16(4):546. doi: 10.3390/biom16040546 (PMC13113681; doi:10.3390/biom16040546)
Supplement: Supplementary file 1 [file biomolecules-16-00546-s001.zip › biomolecules-4142185-supplementary.pdf]

SUPPLEMENTARY FILE

Supplementary Table S1. Plasmids (accession number and amino acids sequence of our constructs)

NAME	Plasmid	Accession number
GFP	pET28a	P42212
GFP-ApoAI	pET28a	P42212<br>NP_000030.1
GFP-EmrE-ApoAI	pET28a	P42212<br>P23895<br>NP_000030.1
GFP-ApoaI-EmrE-CPP	pET28a	P42212<br>NP_000030.1<br>P23895
TatA	pET28a	P69698
TatAx1	pET28a	P69698
TatAx3	pET28a	P69698
OspA-TatAx3-ApoAI	pET28a	WP_259023821.1<br>P69698<br>NP_000030.1
MBP-TatAx3-ApoAI	pET28a	P0AEX9<br>P69698<br>NP_000030.1
GFP-ApoAI-TatAx3-CPP	pET28a	P42212<br>NP_000030.1<br>P69698
OspA-ApoAI-cahFZD7-CPP	pET28a	WP_259023821.1<br>NP_000030.1<br>O75084
OspA-ApoAI-cahGPR56-CPP	pET28a	WP_259023821.1<br>NP_000030.1<br>Q9Y653
OspA-ApoAI-capFZD4-CPP	pET28a	WP_259023821.1<br>NP_000030.1<br>ABW97516.1
OspA-ApoAI-capFZD1/2/7-CPP	pET28a	WP_259023821.1<br>NP_000030.1<br>AQT19762.1
OspA-ApoAI-MthK-CPP	pET28a	WP_259023821.1<br>NP_000030.1<br>O27564

TATAx1

SKHMGGISIW QLLIIAVIVV LLFGTKKLGS IGSDLGASIK GFKKAMSEQK LISEEDL

TATAx3

SKHMGGISIW QLLIIAVIVV LLFGTKKLGS IGSDLGASIK GFKKAMSEQK LISEEDL
MGG ISIWQLLIIA VIVVLLFGTK KLGSIGSDLG ASIKGFKKAM SEQKLISEED L
MGGISIWQL LIIAVIVVLL FGTKKLGSIG SDLGASIKGF KKAMSDDEPK QDKEF

OspA-TatAx3-ApoAI

10 20 30 40 50 60
MGNSVSVDLP GSMKVLVSKS SNADGKYDLI ATVDALELSG TSDKNNGSGV LEGVKADASK

70 80 90 100 110 120
VKLTISDDLG QTTLEVFKSD GSTLVSKKVT SKHMGGISIW QLLIIAVIVV LLFGTKKLGS

130 140 150 160 170 180
IGSDLGASIK GFKKAMSEQK LISEEDLMGG ISIWQLLIIA VIVVLLFGTK KLGSIGSDLG

190 200 210 220 230 240
ASIKGFKKAM SEQKLISEED LMGGISIWQL LIIAVIVVLL FGTKKLGSIG SDLGASIKGF

250 260 270 280 290 300
KKAMSDDEPK QDKEFHTLKL LDNWDSVTST FSKLREQLGP VTQEFWDNLE KETEGLRQEM

310 320 330 340 350 360
SKDLEEVKAK VQPYLDDFQK KWQEEMELYR QKVEPLRAEL QEGARQKLHE LQEKLSPLGE

370 380 390 400 410 420
EMRDRARAHV DALRTHLAPY SDELRQRLAA RLEALKENGG ARLAEYHAKA TEHLSTLSEK

430 440 450 460
AKPALEDLRQ GLLPVLESFK VSFLSALEEY TKKLNTQAAA LEHHHHHH

OspA-ApoAI-TatAx3-CPP

10 20 30 40 50 60
MGSSHHHHHH GSGNSVSVDL PGSMKVLVSK SSNADGKYDL IATVDALELS GTSDKNNGSG

70 80 90 100 110 120
VLEGVKADAS KVKLTISDDL GQTTLEVFKS DGSTLVSKKV TSKHTLKLLD NWDSVTSTFS

130 140 150 160 170 180
KLREQLGPVT QEFWDNLEKE TEGLRQEMSK DLEEVKAKVQ PYLDDFQKKW QEEMELYRQK

190 200 210 220 230 240
VEPLRAELQE GARQKLHELQ EKLSPLGEEM RDRARAHVDA LRTHLAPYSD ELRQRLAARL

250 260 270 280 290 300
EALKENGGAR LAEYHAKATE HLSTLSEKAK PALEDLRQGL LPVLESFKVS FLSALEEYTK

310 320 330 340 350 360
KLNTQAAAHM GGISIWQLLI IAVIVVLLFG TKKLGSIGSD LGASIKGFKK AMSEQKLISE

370 380 390 400 410 420
EDLMGGISIW QLLIIAVIVV LLFGTKKLGS IGSDLGASIK GFKKAMSEQK LISEEDLMGG

430 440 450 460 470 480
ISIWQLLIIA VIVVLLFGTK KLGSIGSDLG ASIKGFKKAM SDDEPKQDKG SGKKGSGRRG

SGKK

OspA-ApoAI-cahFZD7-CPP

```
        10         20         30         40         50         60
MGSSHHHHHH SSGNSVSVDL PGSMKVLVSK SSNADGKYDL IATVDALELS GTSDKNNGSG

        70         80         90        100        110        120
VLEGVKADAS KVKLTISDDL GQTTLEVFKS DGSTLVSKKV TSKAAAHTLK LLDNWDSVTS

       130        140        150        160        170        180
TFSKLREQLG PVTQEFWDNL EKETEGLRQE MSKDLEEVKA KVQPYLDDFQ KKWQEEMELY

       190        200        210        220        230        240
RQKVEPLRAE LQEGARQKLH ELQEKLSPLG EEMRDRARAH VDALRTHLAP YSDELRQRLA

       250        260        270        280        290        300
ARLEALKENG GARLAEYHAK ATEHLSTLSE KAKPALEDLR QGLLPVLESF KVSFLSALEE

       310        320        330        340        350        360
YTKKLNTQSG RFARLWVGVW SVLCCASTLF TVLTYLVDMR RFSYPERPII FLSGCYFMVA

       370        380        390        400        410        420
VAHVAGFLLE DRAVCVERFS DDGYRTVAQG TKKEGCTILF MVLYFFGMAS SIWWVILSLT

       430        440        450        460        470        480
WFLAAGMKWG HEAIEANSQY FHLAAWAVPA VKTITILAMG QVDGDLLSGV CYVGLSSVDA

       490        500        510        520        530        540
LRGFVLAPLF VYLFIGTSFL LAGFVSLFRI RTIMKHAGTK TEKLEALMVR IGVFSVLYTV

       550        560        570        580        590        600
PATIVLACYF YEQAFREHWE RTWLLQTCKS YAVPCPPGHF PPMSPDFTVF MIKYLMTMIV

       610        620        630        640
GITTGFWIWS GKTLQSWRRF YHRLSHSSKG ETAVGSGKKG SGRRGSGKK
```

OspA-ApoAI-cahGPR56-CPP

```
        10         20         30         40         50         60
MGSSHHHHHH SAAAGGNSVS VDLPGSMKVL VSKSSNADGK YDLIATVDAL ELSGTSDKNN

        70         80         90        100        110        120
GSGVLEGVKA DASKVKLTIS DDLGQTTLEV FKSDGSTLVS KKVTSKAAAH TLKLLDNWDS

       130        140        150        160        170        180
VTSTFSKLRE QLGPVTQEFW DNLEKETEGL RQEMSKDLEE VKAKVQPYLD DFQKKWQEEM

       190        200        210        220        230        240
ELYRQKVEPL RAELQEGARQ KLHELQEKLS PLGEEMRDRA RAHVDALRTH LAPYSDELRQ

       250        260        270        280        290        300
RLAARLEALK ENGGARLAEY HAKATEHLST LSEKAKPALE DLRQGLLPVL ESFKVSFLSA

       310        320        330        340        350        360
LEEYTKKLNT QGSGPKNVTL QCVFWVEDPT LSSPGHWSSA GCETVRRETQ TSCFCNHLTY

       370        380        390        400        410        420
FAVLMVSSVE VDAVHKHYLS LLSYVGCVVS ALACLVTIAA YLCSRVPLPC RRKPRDYTIK

       430        440        450        460        470        480
VHMNLLLAVF LLDTSFLLSE PVALTGSEAG CRASAIFLHF SLLTCLSWMG LEGYNLYRLV

       490        500        510        520        530        540
VEVFGTYVPG YLLKLSAMGW GFPIFLVTLV ALVDVDNYGP IILAVHRTPE GVIYPSMCWI

       550        560        570        580        590        600
```

```
RDSLVSYITN LGLFSLVFLF NMAMLATMVV QILRLRPHTQ KWSHVLTLLG LSLVLGLPWA

       610        620        630        640        650        660
LIFFSFASGT FQLVVLYLFS IITSFQGFLI FIWYWSMRLQ ARRGSGKKGS GKKGSGRRGS

GKK
```

OspA-ApoAI-capFZD4-CPP

```
        10         20         30         40         50         60
MGSSHHHHHH SSGNSVSVDL PGSMKVLVSK SSNADGKYDL IATVDALELS GTSDKNNGSG

        70         80         90        100        110        120
VLEGVKADAS KVKLTISDDL GQTTLEVFKS DGSTLVSKKV TSKAAAHTLK LLDNWDSVTS

       130        140        150        160        170        180
TFSKLREQLG PVTQEFWDNL EKETEGLRQE MSKDLEEVKA KVQPYLDDFQ KKWQEEMELY

       190        200        210        220        230        240
RQKVEPLRAE LQEGARQKLH ELQEKLSPLG EEMRDRARAH VDALRTHLAP YSDELRQRLA

       250        260        270        280        290        300
ARLEALKENG GARLAEYHAK ATEHLSTLSE KAKPALEDLR QGLLPVLESF KVSFLSALEE

       310        320        330        340        350        360
YTKKLNTQSG HDKVFANIWL LGWSVLCFFS CLLTIIVFSC NTNRFLYPEK PIVFLSICYF

       370        380        390        400        410        420
FYASGNLFGA ILGRNVVACR AFNDKTDFIV GSGRETTWCK INFLMIYFFG SASALWWVVL

       430        440        450        460        470        480
TITWFLSASR HWGYEAIESI SSMLHLVSWA IPALKSIFIL ILHKIDADEL TGQCFVGNSN

       490        500        510        520        530        540
NKVLWGFVIV PNMIYLFIGI VFLTMGYISL LKVRRSFLQR PDCIATNNLK RLAKLMAKIG

       550        560        570        580        590        600
VFSILYVLPV LCTIVSYIVD AYKMTNFDIT LKALFRFSPN CIGRNGINWS SIKCVKILQP

       610        620        630        640        650        660
VLPSVEMRML RIFMNLVIGI TSGIWIWGNK KTVKSCISTL RFWEKKKEEE PTPTNQCDSS

       670        680        690        700        710
TIQNNIPLSQ TKQKETLLPY HDWKLNSSYP VVVEYGLPTV SVPGSGKKGS GRRGSGKK
```

OspA-ApoAI-capFZD1/2/7

```
        10         20         30         40         50         60
MGSSHHHHHH SSGNSVSVDL PGSMKVLVSK SSNADGKYDL IATVDALELS GTSDKNNGSG

        70         80         90        100        110        120
VLEGVKADAS KVKLTISDDL GQTTLEVFKS DGSTLVSKKV TSKAAAHTLK LLDNWDSVTS

       130        140        150        160        170        180
TFSKLREQLG PVTQEFWDNL EKETEGLRQE MSKDLEEVKA KVQPYLDDFQ KKWQEEMELY

       190        200        210        220        230        240
RQKVEPLRAE LQEGARQKLH ELQEKLSPLG EEMRDRARAH VDALRTHLAP YSDELRQRLA

       250        260        270        280        290        300
ARLEALKENG GARLAEYHAK ATEHLSTLSE KAKPALEDLR QGLLPVLESF KVSFLSALEE

       310        320        330        340        350        360
YTKKLNTQSG RKFARVWVAL WSFMCVGSTL FTVLTFLIDM KRFQYPERPI IFLSACYLVV

       370        380        390        400        410        420
```

GLTYVAGFFL NDKVACAGPF TNKDSTGKTP KIVVQGTKFE SCIILFMLLY FFSMASAIWW

430 440 450 460 470 480
VVLTITWYLA AKCHWAHESI GRNSQYFHFA AWAIPAGKTI GILALSKVDG DPLTGVCFTG

490 500 510 520 530 540
LSDPSTLRGF LIAPLCIYLL IGTGFLIAGF VSMFEIRTII KTAGSKTDKL AKLITRIGIF

550 560 570 580 590 600
SVLYVVPAVV VIACYFHESM NLYKWMQRWY LIDICRSAEY KDECKKIHDH KPINPGGNAL

610 620 630 640 650 660
IFSQKPEFEL FMIKYLMSLI VGITSGIWIW SGKTLLSWKY FFSRLCGRLN PGDPWTRSDW

670 680 690 700 710 720
LEKNNGYYQR HFVGPTANKM IIQGCDDKQA IPLQYQFPQQ TQQTIGTQEL QKPLLSNVPE

730 740 750 760
SILNAQAQNG NLLPPSQQIG DSKTQKIGFM MGSGKKGSGR RGSGKK

OspA-ApoAI-MthK-CPP

10 20 30 40 50 60

MGSSHHHHHH SSGNSVSVDL PGSMKVLVSK SSNADGKYDL IATVDALELS GTSDKNNGSG

70 80 90 100 110 120
VLEGVKADAS KVKLTISDDL GQTTLEVFKS DGSTLVSKKV TSKAAAHTLK LLDNWDSVTS

130 140 150 160 170 180
TFSKLREQLG PVTQEFWDNL EKETEGLRQE MSKDLEEVKA KVQPYLDDFQ KKWQEEMELY

190 200 210 220 230 240
RQKVEPLRAE LQEGARQKLH ELQEKLSPLG EEMRDRARAH VDALRTHLAP YSDELRQRLA

250 260 270 280 290 300
ARLEALKENG GARLAEYHAK ATEHLSTLSE KAKPALEDLR QGLLPVLESF KVSFLSALEE

310 320 330 340 350 360
YTKKLNTQSG VLVIEIIRKH LPRVLKVPAT RILLLVLAVI IYGTAGFHFI EGESWTVSLY

370 380 390 400 410 420
WTFVTIATVG YGDYSPSTPL GMYFTVTLIV LGIGTFAVAV ERLLEFLINR EQMKLMGLID

430 440 450 460 470 480
VAKSRHVVIC GWSESTLECL RELRGSEVFV LAEDENVRKK VLRSGANFVH GDPTRVSDLE

490 500 510 520 530 540
KANVRGARAV IVDLESDSET IHCILGIRKI DESVRIIAEA ERYENIEQLR MAGADQVISP

550 560 570 580 590 600
FVISGRLMSR SIDDGYEAMF VQDVLAEEST RRMVEVPIPE GSKLEGVSVL DADIHDVTGV

610 620 630 640 650 660
IIIGVGRGDE LIIDPPRDYS FRAGDIILGI GKPEEIERLK NYISAGSGKK GSGRRGSGKK

Figure SI1. Epigenetics Sample metadata

Path	Sample_Name	Sample_Type	Treatment	Slide	Array
./Data/207096530008_R01C01	207096530008_R01C01	HEK	Control (RA)	Aston_MethylationEPICv2_20230426	EPICv2
./Data/207096530008_R02C01	207096530008_R02C01	HEK	Control (RA)	Aston_MethylationEPICv2_20230426	EPICv2
./Data/207096530008_R03C01	207096530008_R03C01	HEK	CHIR99021+RA	Aston_MethylationEPICv2_20230426	EPICv2
./Data/207096530008_R04C01	207096530008_R04C01	HEK	CHIR99021+RA	Aston_MethylationEPICv2_20230426	EPICv2
./Data/207096530008_R05C01	207096530008_R05C01	HEK	iDRIVEcapFZD7+RA	Aston_MethylationEPICv2_20230426	EPICv2
./Data/207096530008_R06C01	207096530008_R06C01	HEK	iDRIVEcapFZD7+RA	Aston_MethylationEPICv2_20230426	EPICv2

R Session information

R version 4.5.1 (2025-06-13)
Platform: aarch64-apple-darwin20
Running under: macOS Tahoe 26.2

Matrix products: default
BLAS:
/System/Library/Frameworks/Accelerate.framework/Versions/A/Frameworks/vecLib.framework/Versions/A/libBLAS.dylib
LAPACK: /Library/Frameworks/R.framework/Versions/4.5-arm64/Resources/lib/libRlapack.dylib; LAPACK version 3.12.1

locale:
[1] en_US.UTF-8/en_US.UTF-8/en_US.UTF-8/C/en_US.UTF-8/en_US.UTF-8

time zone: America/Denver
tzcode source: internal

attached base packages:
 [1] grid   parallel stats4   stats   graphics grDevices utils   datasets methods   base

other attached packages:
 [1] lumi_2.62.0   ggVennDiagram_1.5.7
 [3] eulerr_7.0.4   ComplexHeatmap_2.26.1
 [5] RColorBrewer_1.1-3   pheatmap_1.0.13
 [7] ggplot2_4.0.2   dplyr_1.2.0
 [9] sva_3.58.0   BiocParallel_1.44.0

```
[11] genefilter_1.92.0            mgcv_1.9-4
[13] nlme_3.1-168                 DMRcate_3.6.0
[15] limma_3.66.0                 IlluminaHumanMethylationEPICv2manifest_1.0.1
[17] minfi_1.56.0                 bumphunter_1.52.0
[19] locfit_1.5-9.12              iterators_1.0.14
[21] foreach_1.5.2                Biostrings_2.78.0
[23] XVector_0.50.0               SummarizedExperiment_1.40.0
[25] Biobase_2.70.0               MatrixGenerics_1.22.0
[27] matrixStats_1.5.0            GenomicRanges_1.62.1
[29] Seqinfo_1.0.0                IRanges_2.44.0
[31] S4Vectors_0.48.0             BiocGenerics_0.56.0
[33] generics_0.1.4

loaded via a namespace (and not attached):
  [1] ProtGenerics_1.42.0                    bitops_1.0-9
  [3] httr_1.4.8                             doParallel_1.0.17
  [5] tools_4.5.1                            doRNG_1.8.6.3
  [7] backports_1.5.0                        R6_2.6.1
  [9] HDF5Array_1.38.0                       Gviz_1.54.0
 [11] lazyeval_0.2.2                         rhdf5filters_1.22.0
 [13] GetoptLong_1.1.0                       permute_0.9-10
 [15] methylumi_2.56.0                       withr_3.0.2
 [17] prettyunits_1.2.0                      gridExtra_2.3
 [19] base64_2.0.2                           preprocessCore_1.72.0
 [21] cli_3.6.5                              labeling_0.4.3
 [23] S7_0.2.1
IlluminaHumanMethylationEPICanno.ilm10b4.hg19_0.6.0
 [25] readr_2.2.0                            askpass_1.2.1
 [27] Rsamtools_2.26.0                       foreign_0.8-91
 [29] siggenes_1.84.0                        illuminaio_0.52.0
 [31] R.utils_2.13.0                         rentrez_1.2.4
 [33] dichromat_2.0-0.1                      scrime_1.3.7
 [35] BSgenome_1.78.0                        rstudioapi_0.18.0
 [37] RSQLite_2.4.6                          shape_1.4.6.1
 [39] BiocIO_1.20.0                          gtools_3.9.5
 [41] car_3.1-5                              interp_1.1-6
 [43] Matrix_1.7-4                           abind_1.4-8
 [45] R.methodsS3_1.8.2                      lifecycle_1.0.5
 [47] yaml_2.3.12                            edgeR_4.8.2
 [49] carData_3.0-6                          rhdf5_2.54.1
 [51] SparseArray_1.10.8                     BiocFileCache_3.0.0
 [53] blob_1.3.0                             ExperimentHub_3.0.0
 [55] crayon_1.5.3                           lattice_0.22-9
 [57] beachmat_2.26.0                        GenomicFeatures_1.62.0
 [59] cigarillo_1.0.0                        annotate_1.88.0
```

```
 [61] KEGGREST_1.50.0                            pillar_1.11.1
 [63] knitr_1.51                              beanplot_1.3.1
 [65] rjson_0.2.23                             codetools_0.2-20
 [67] glue_1.8.0                              data.table_1.18.2.1
 [69] vctrs_0.7.1                             png_0.1-8
 [71] gtable_0.3.6
IlluminaHumanMethylation450kanno.ilmn12.hg19_0.6.1
 [73] cachem_1.1.0                            xfun_0.56
 [75] S4Arrays_1.10.1                          survival_3.8-6
 [77] statmod_1.5.1                           bit64_4.6.0-1
 [79] bsseq_1.46.0                            progress_1.2.3
 [81] filelock_1.0.3                          GenomeInfoDb_1.46.2
 [83] nor1mix_1.3-3                           affyio_1.80.0
 [85] KernSmooth_2.23-26                         otel_0.2.0
 [87] rpart_4.1.24                            Hmisc_5.2-5
 [89] colorspace_2.1-2                         DBI_1.3.0
 [91] nnet_7.3-20                             tidyselect_1.2.1
 [93] bit_4.6.0                              compiler_4.5.1
 [95] curl_7.0.0                             httr2_1.2.2
 [97] htmlTable_2.4.3                          h5mread_1.2.1
 [99] xml2_1.5.2                             DelayedArray_0.36.0
[101] rtracklayer_1.70.1                         checkmate_2.3.4
[103] scales_1.4.0                            affy_1.88.0
[105] quadprog_1.5-8                           rappdirs_0.3.4
[107] stringr_1.6.0                           digest_0.6.39
[109] rmarkdown_2.30                            GEOquery_2.78.0
[111] jpeg_0.1-11                             htmltools_0.5.9
[113] pkgconfig_2.0.3                          base64enc_0.1-6
[115] sparseMatrixStats_1.22.0                    dbplyr_2.5.2
[117] fastmap_1.2.0                           ensembldb_2.34.0
[119] rlang_1.1.7                             GlobalOptions_0.1.3
[121] htmlwidgets_1.6.4                         UCSC.utils_1.6.1
[123] DelayedMatrixStats_1.32.0                   farver_2.1.2
[125] jsonlite_2.0.0                          mclust_6.1.2
[127] R.oo_1.27.1                             VariantAnnotation_1.56.0
[129] RCurl_1.98-1.17                          magrittr_2.0.4
[131] Formula_1.2-5                           Rhdf5lib_1.32.0
[133] Rcpp_1.1.1                              stringi_1.8.7
[135] nleqslv_3.3.6                           MASS_7.3-65
[137] AnnotationHub_4.0.0                        plyr_1.8.9
[139] org.Hs.eg.db_3.22.0                        deldir_2.0-4
[141] splines_4.5.1                           multtest_2.66.0
[143] hms_1.1.4                               circlize_0.4.17
[145] ggpubr_0.6.3                            ggsignif_0.6.4
[147] rngtools_1.5.2                           biomaRt_2.66.1
```

```
[149] BiocVersion_3.22.0              missMethyl_1.44.0
[151] XML_3.99-0.22                   evaluate_1.0.5
[153] latticeExtra_0.6-31             biovizBase_1.58.0
[155] BiocManager_1.30.27             tzdb_0.5.0
[157] tweenr_2.0.3                    tidyr_1.3.2
[159] openssl_2.3.5                   purrr_1.2.1
[161] polyclip_1.10-7                 reshape_0.8.10
[163] clue_0.3-67                     ggforce_0.5.0
[165] broom_1.0.12                    xtable_1.8-8
[167] restfulr_0.0.16                 AnnotationFilter_1.34.0
[169] rstatix_0.7.3                   tibble_3.3.1
[171] memoise_2.0.1                   AnnotationDbi_1.72.0
[173] GenomicAlignments_1.46.0        cluster_2.1.8.2
```

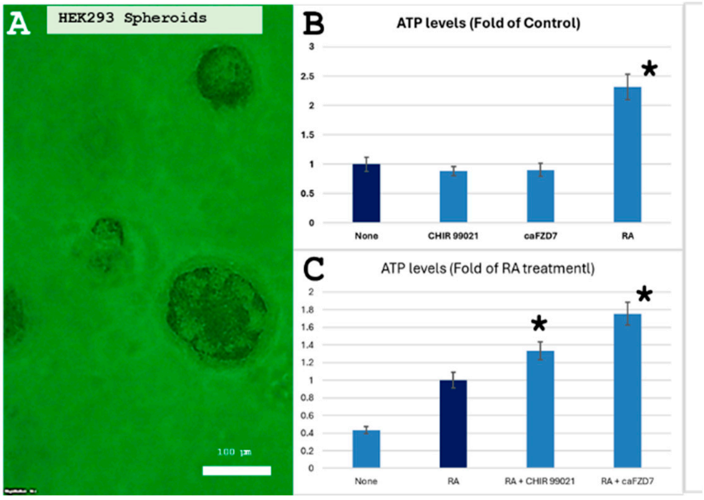

Figure SI2. iDRIVE-Frizzled + RA stimulate HEK 293 embryoid body bioenergetics.

A) Imaging HEK 293 cells cultured as Embryoid Bodies (EBs). **B)** ATP assay of HEK 293 cells in culture. Cells are exposed to CHIR99021, iDRIVE (caFZD7), or Retinoic Acid (RA). Dark blue is used for None (no treatment) and is the control over which ATP levels are considered. **C)** Combinations of CHIR99021 and iDRIVE with RA were tested. Dark blue is used for RA only and is the control over which ATP levels are considered. Asterisks represent statistically significant differences against the control ($p<0.04$). n = 48 per treatment group.

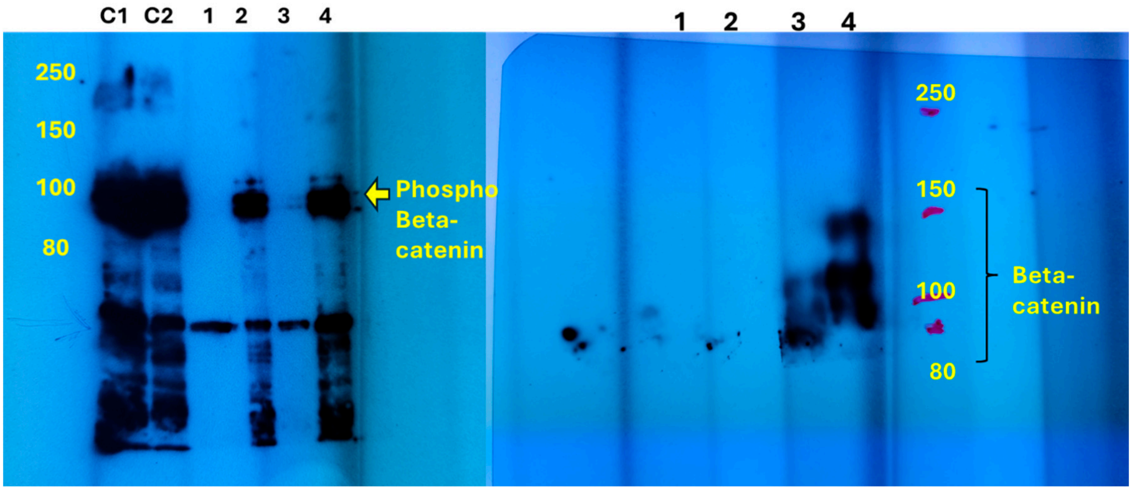

Figure SI3. Phospho-β-catenin and β-catenin western blot. Santa Cruz Biotechnologies, Left panel, Phosphorylated-beta-catenin Antibody (1B11): sc-57533; C1 and C2 - HEK293 cells untreated, 1-Planaria tail+iDRIVE capFRZD1/2/7; 2- Planaria tail+control protein; 3-Planaria head+Control protein; 4-Planaria tail+control protein. **Right panel,** Beta-catenin Antibody (12F7): sc-59737. Antibodies are used 1:250 dilution. Incubated at 4 C overnight. Secondary antibodies are diluted 1:3,000. 1 and 2-Planaria tail+control protein; 2- Planaria tail+iDRIVE capFRZD1/2/7 protein; 3-Planaria head+control protein. Numbers correspond to Size Markers (kDa).

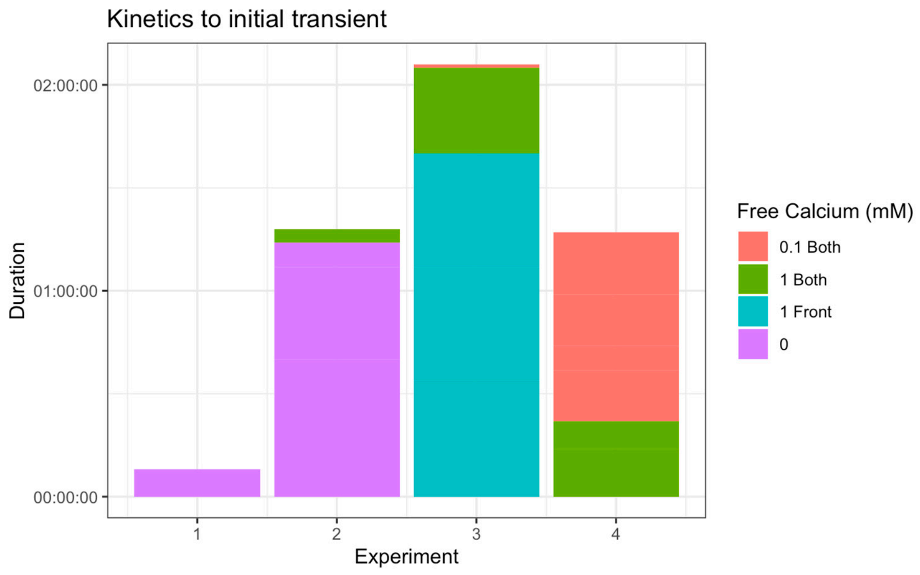

Figure SI4. MthK incubation and free calcium concentration.

Free calcium concentration in PLB experiments. The first conductance event for experiment 3 occurred immediately after the application of 2 mM EDTA (cis and trans chambers). This may suggest an RCK conformational change that is not conducive to plasma membrane insertion.

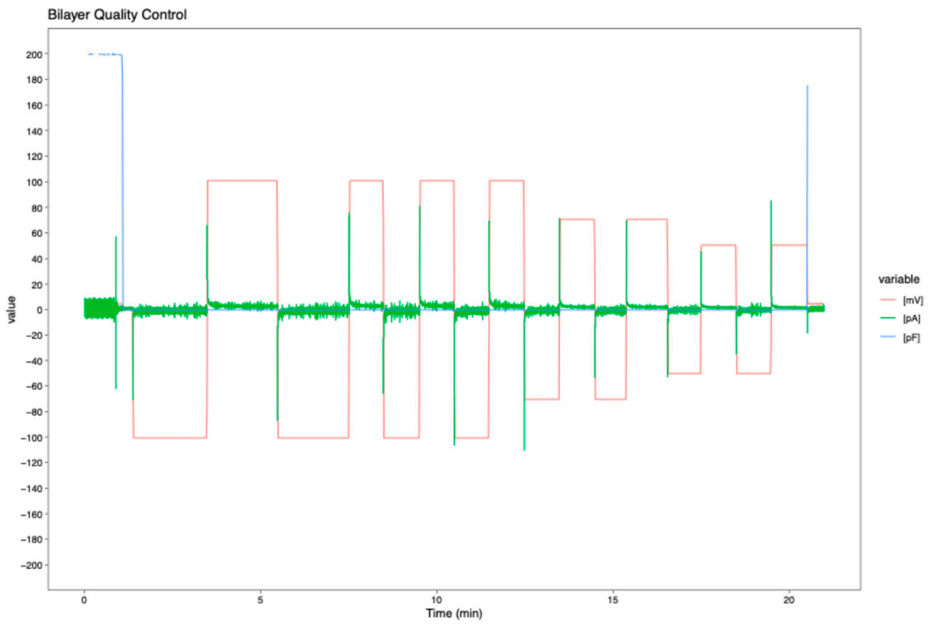

Figure SI5. Planar bilayer conductance quality control.

Alternating voltages between 100 mV and -100 mV applied intermittently every 1-2 minutes. Lipid bilayer integrity assessed through capacitance (pF) measurements.

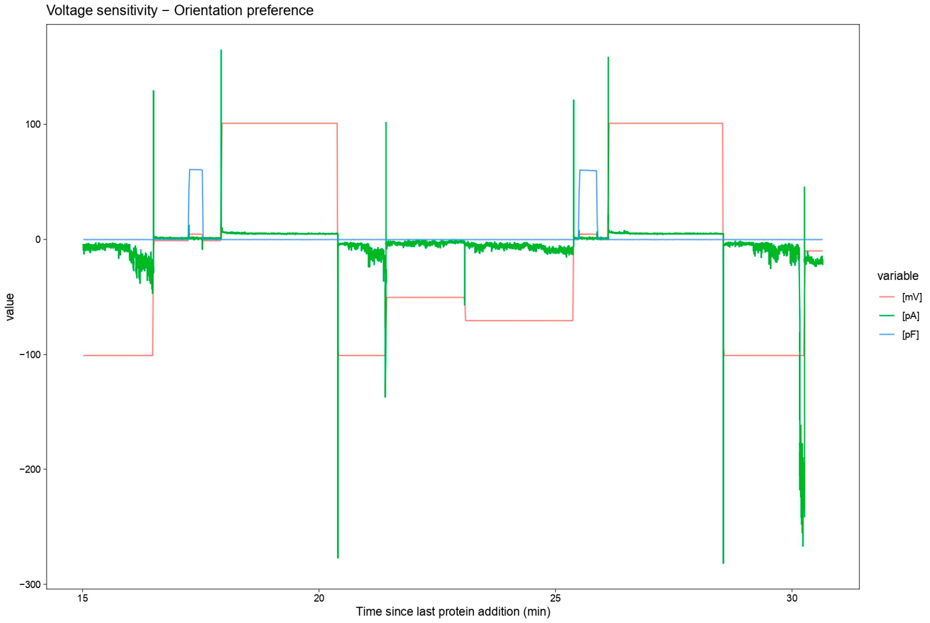

Figure SI6. iDRIVE-MthK re-inserts the lipid bilayer via its C-terminus.

Extension of Figure 5C. Conductance is only triggered with negative voltages, suggesting iDRIVE mediates the insertion into plasma membranes in a uniform orientation. See § 3.4 in the main article for further discussion.

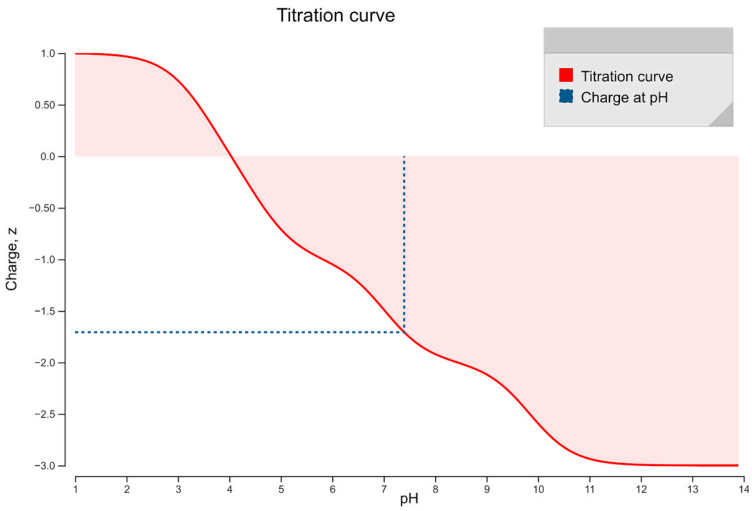

Figure SI7. MthK selectivity filter Titration Curve.

Selectivity filter (TVGYGD) for each MthK subunit was predicted to have a charge of ~ -1.9 at pH 8.02. Thus, we rule out anion currents through MthK.

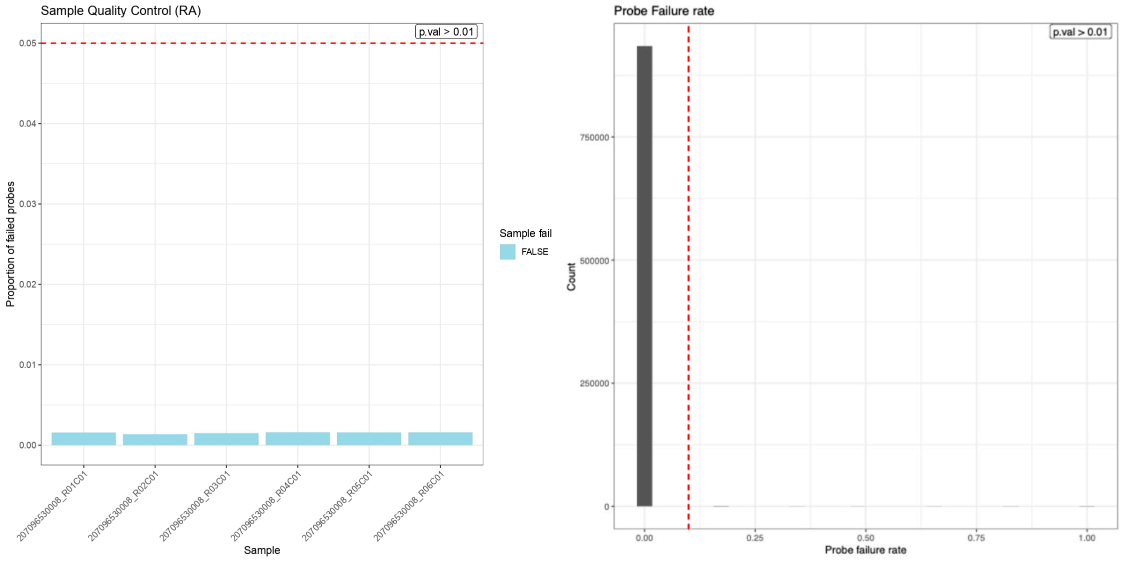

Figure SI8. EPICv2 array sample and probe quality control.

Probes with detection p-val > 0.01 were considered "failed." Sample quality control was performed by filtering samples that had over 5% "failed" probes. Similarly, probe filtering was performed by removing probes that "failed" in over 10% of the samples.

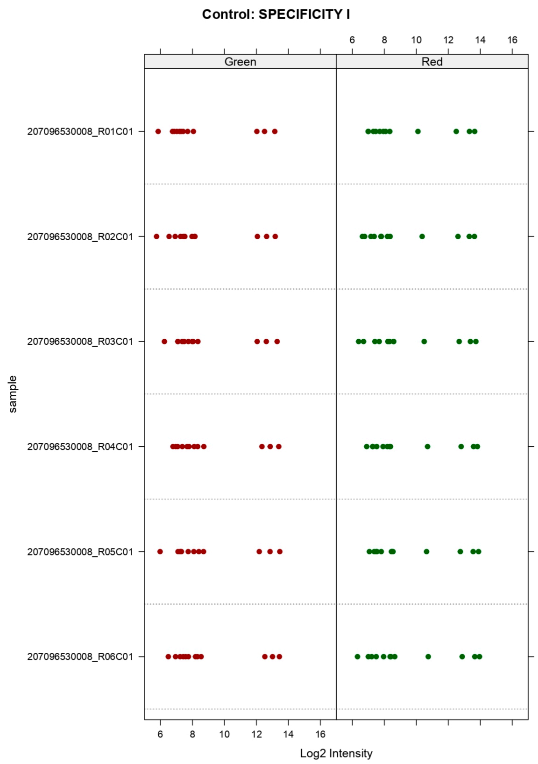
Control: SPECIFICITY I
Green
Red
sample
207096530008_R01C01
207096530008_R02C01
207096530008_R03C01
207096530008_R04C01
207096530008_R05C01
207096530008_R06C01
6 8 10 12 14 16
Log2 Intensity

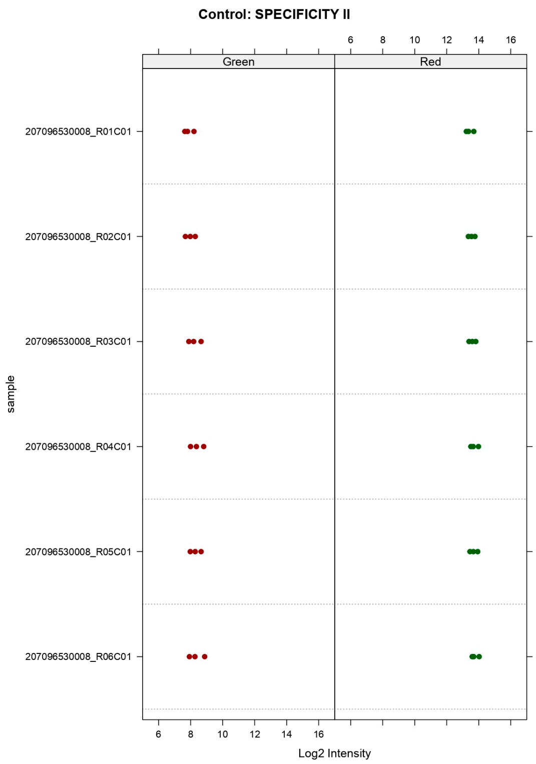
Control: SPECIFICITY II
Green
Red
sample
207096530008_R01C01
207096530008_R02C01
207096530008_R03C01
207096530008_R04C01
207096530008_R05C01
207096530008_R06C01
6 8 10 12 14 16
Log2 Intensity

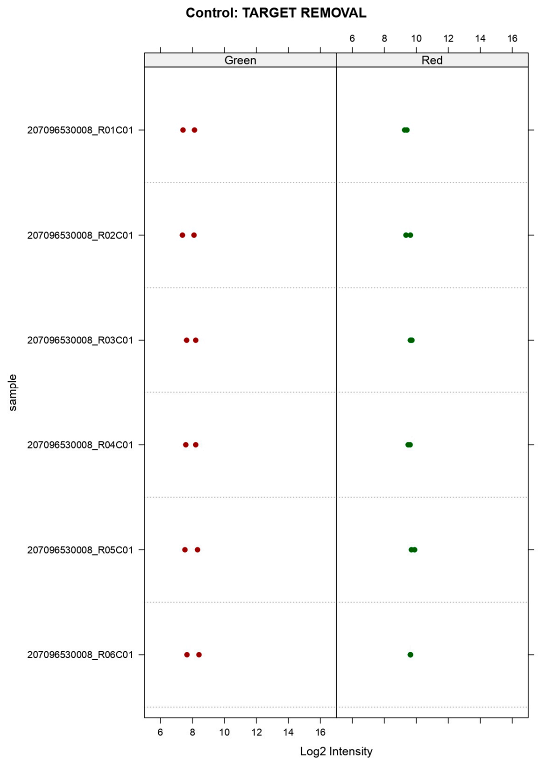
Control: TARGET REMOVAL
Green
Red
sample
207096530008_R01C01
207096530008_R02C01
207096530008_R03C01
207096530008_R04C01
207096530008_R05C01
207096530008_R06C01
6 8 10 12 14 16
Log2 Intensity

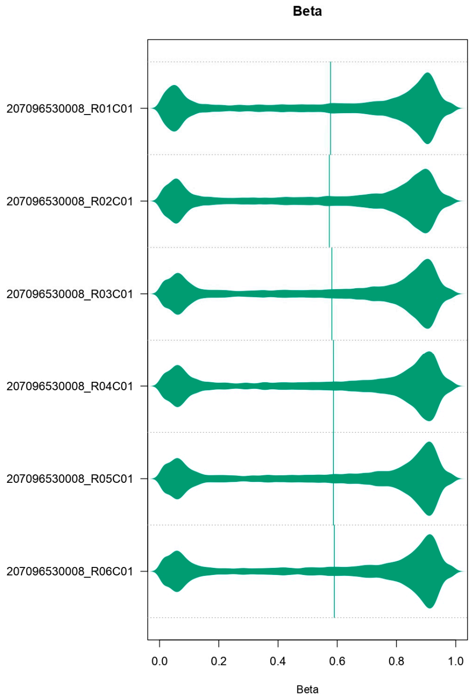
Beta
207096530008_R01C01
207096530008_R02C01
207096530008_R03C01
207096530008_R04C01
207096530008_R05C01
207096530008_R06C01
0.0 0.2 0.4 0.6 0.8 1.0
Beta

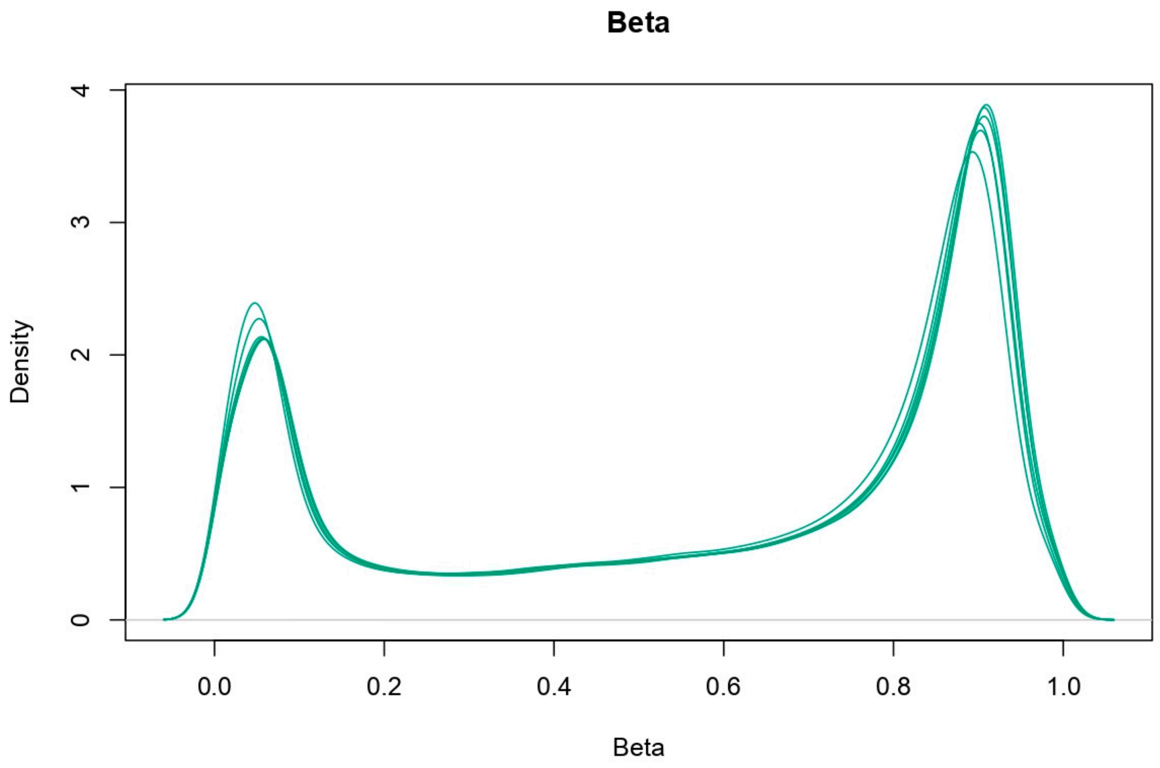
Beta
Density
Beta
0 1 2 3 4
0.0 0.2 0.4 0.6 0.8 1.0

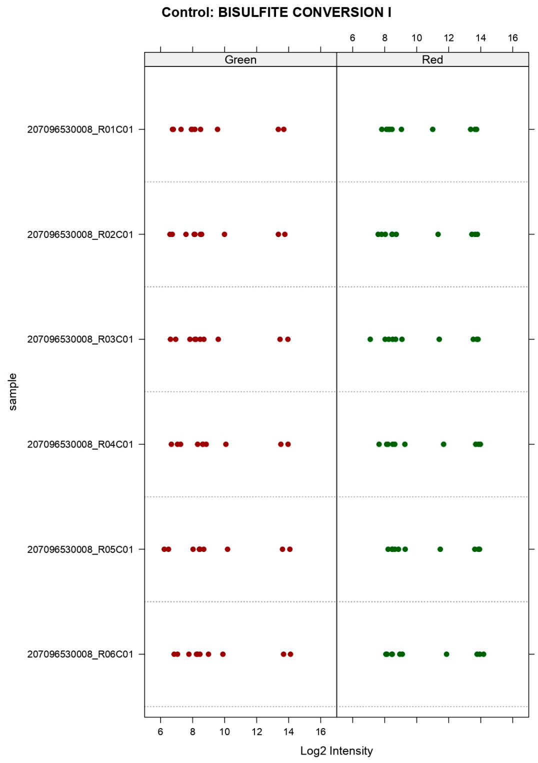
Control: BISULFITE CONVERSION I
Green
Red
207096530008_R01C01
207096530008_R02C01
207096530008_R03C01
207096530008_R04C01
207096530008_R05C01
207096530008_R06C01
sample
Log2 Intensity
6 8 10 12 14 16

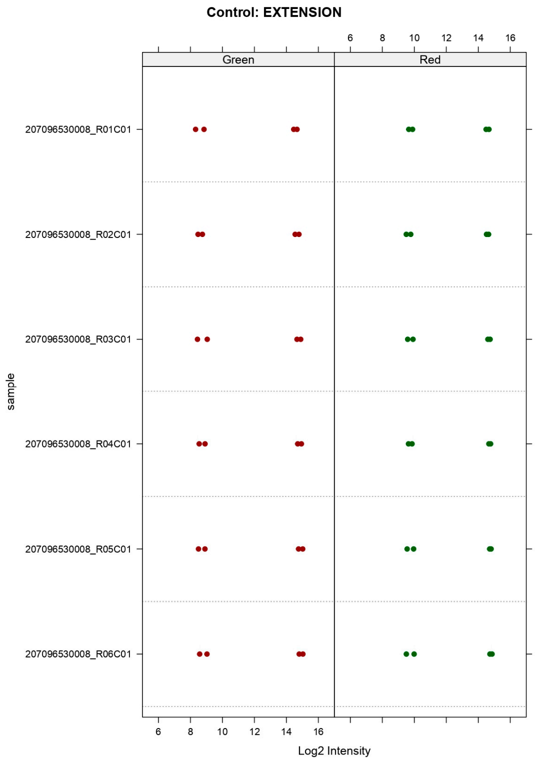
Control: EXTENSION
Green
Red
207096530008_R01C01
207096530008_R02C01
207096530008_R03C01
207096530008_R04C01
207096530008_R05C01
207096530008_R06C01
sample
Log2 Intensity
6 8 10 12 14 16

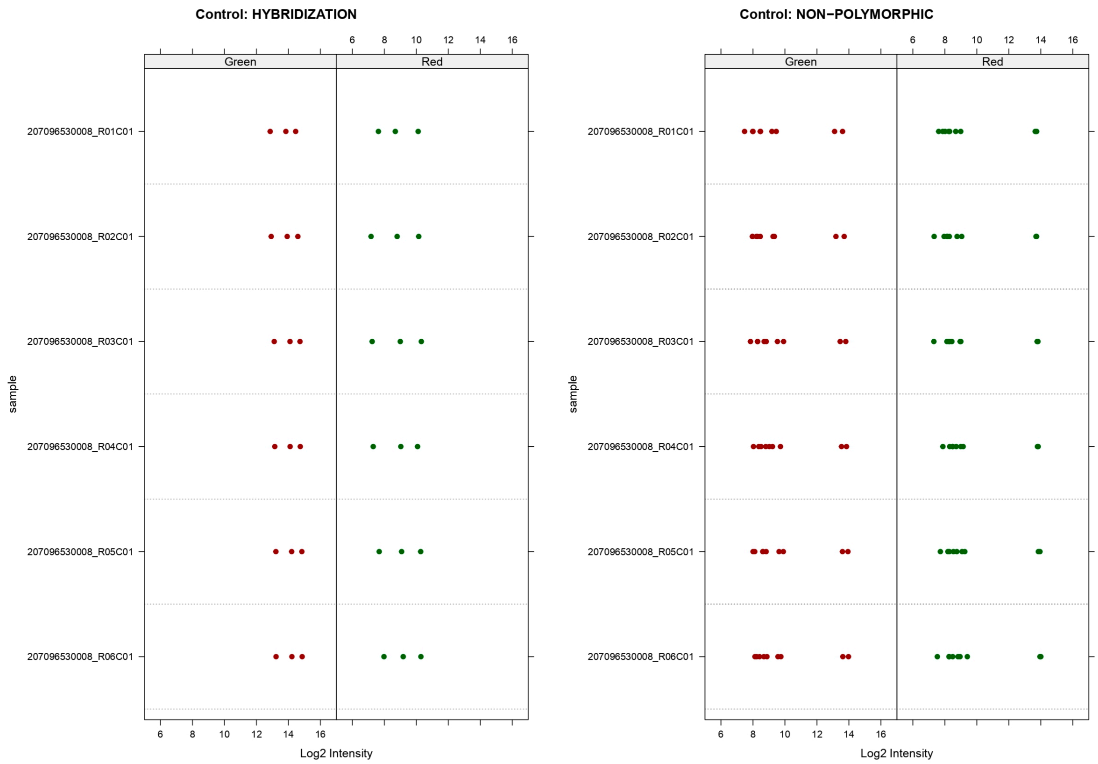

Figure SI9. Comprehensive EPICv2 array methylation quality control.

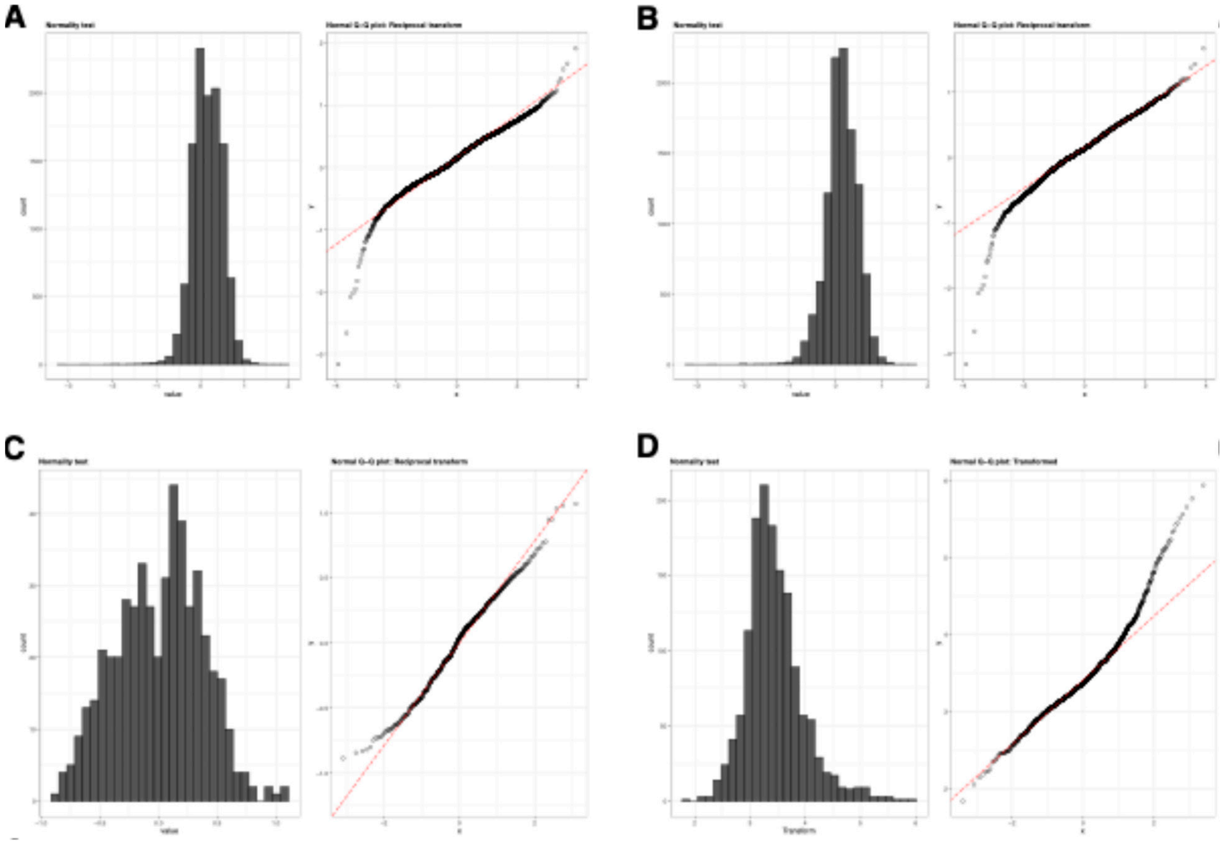

Figure SI10. Normality assessments.

A-C) Average M-value differences across DMRs. **A)** DMRs identified in iDRIVE-cahFZD7 + RA vs RA. **B)** DMRs identified in CHIR99021 + RA vs RA. **C)** DMRs identified in CHIR99021+RA vs iDRIVE-cahFZD7+RA. **D)** Log transformed myotube diameters (Hypertrophy assay)

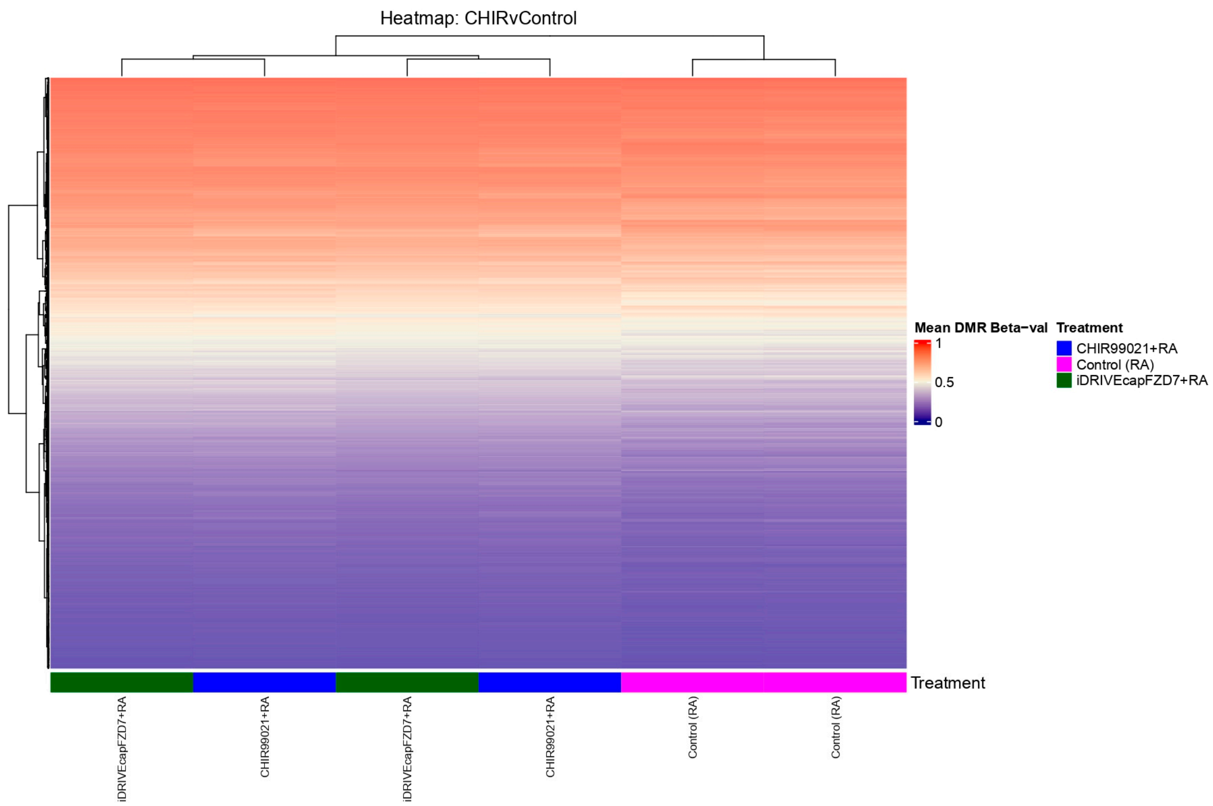

Figure SI11. Average DMR Beta values for significant DMRs in the CHIR99021+RA vs RA contrast.

We note that CHIR99021+RA and iDRIVE-cahFZD7+RA cluster better with each other than with their replicates, L1 distance – average linkage hierarchical clustering.

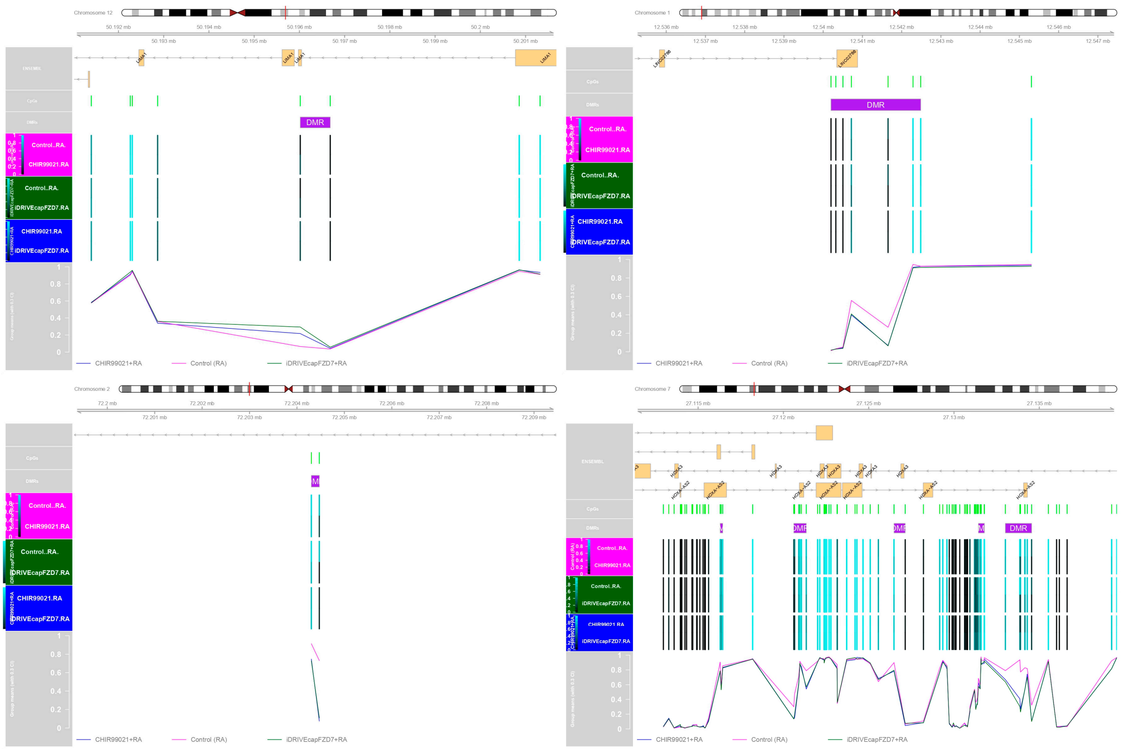

Figure SI12. DMR region plots: iDRIVE-cahFZD7+RA vs Control (RA). Top 4 most significant DMRs found in the contrast. We note the close trends between iDRIVE-cahFZD7+RA and CHIR99021+RA methylation patterns.

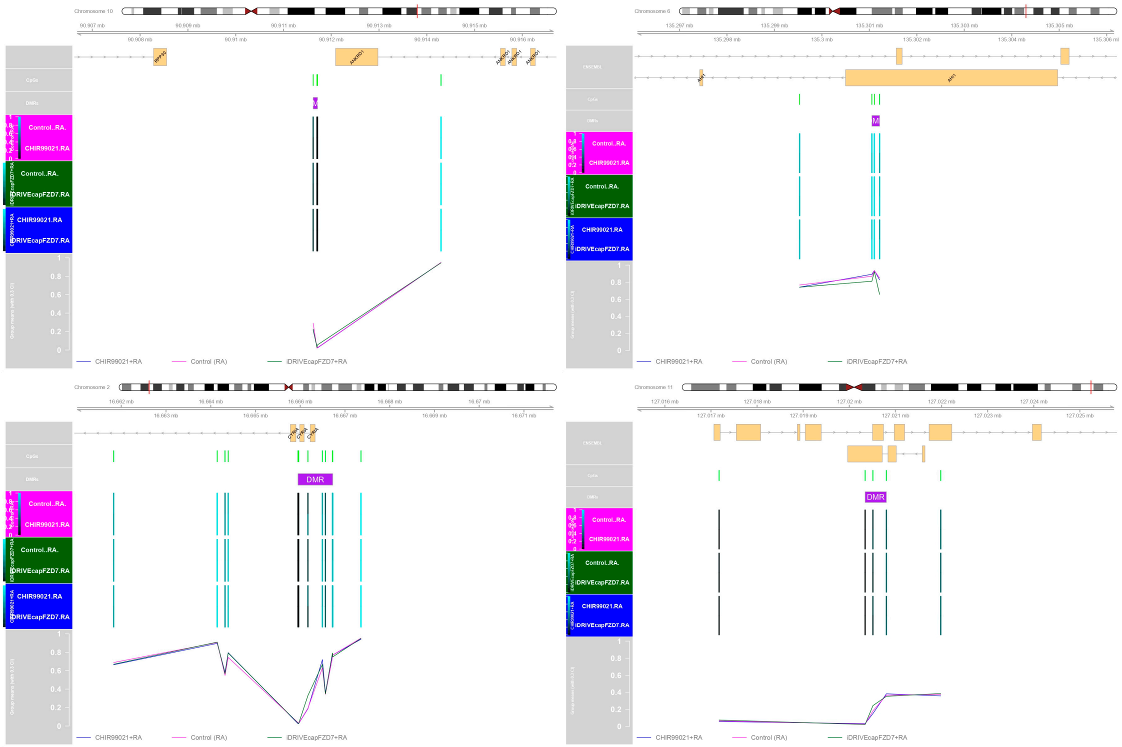

Figure SI13. DMR region plots: iDRIVE-cahFZD7+RA vs CHIR99021+RA.

We note the unique epigenetic patterns elicited by iDRIVE-cahFZD7+RA and not through CHIR99021+RA.

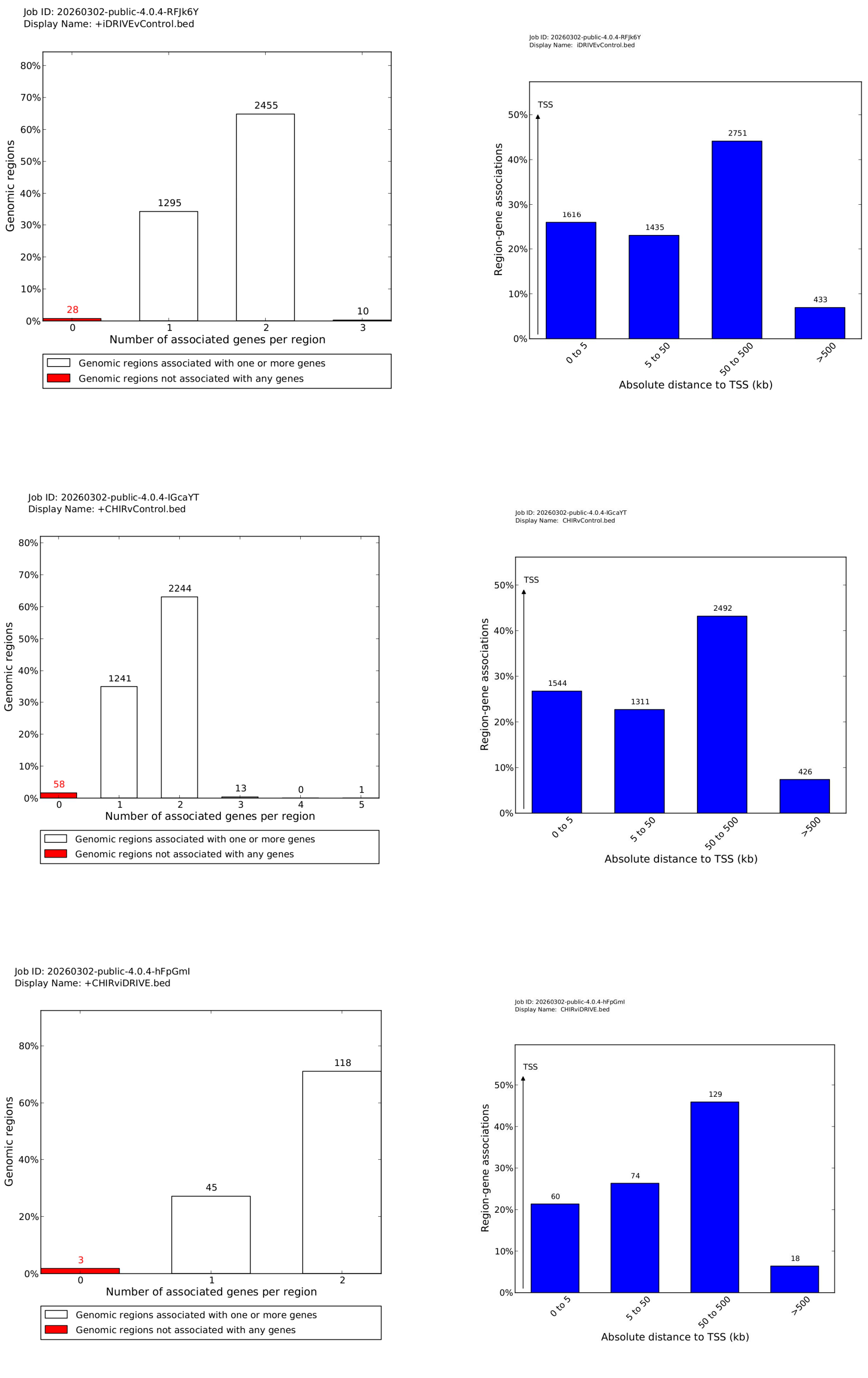

Figure SI14. Associated gene count per DMR and absolute distance to TSS.

We note that iDRIVE and CHIR had a similar number of associated genes with DMRs, but iDRIVE tended to result in higher number of enriched associations. Similar results were observed with absolute distances and distances (data not shown) to TSS.
